# The endosomal system of primary human vascular endothelial cells and albumin–FcRn trafficking

**DOI:** 10.1242/jcs.260912

**Published:** 2023-08-11

**Authors:** Andreas Pannek, Janine Becker-Gotot, Steven K. Dower, Anne M. Verhagen, Paul A. Gleeson

**Affiliations:** ^1^ The Department of Biochemistry and Pharmacology and Bio21 Molecular Science and Biotechnology Institute, The University of Melbourne, Victoria 3010, Australia; ^2^ Institute of Molecular Medicine and Experimental Immunology (IMMEI), University Clinic Bonn, Rheinische Friedrich-Wilhelms-Universität, Venusberg Campus 1, 53127 Bonn, Germany; ^3^ CSL Limited, Research, Bio21 Molecular Science and Biotechnology Institute, Victoria 3010, Australia

**Keywords:** Neonatal Fc receptor, Albumin, Primary endothelial cells, Blood outgrowth endothelial cells, Macropinocytosis, Cargo recycling

## Abstract

Human serum albumin (HSA) has a long circulatory half-life owing, in part, to interaction with the neonatal Fc receptor (FcRn or FCGRT) in acidic endosomes and recycling of internalised albumin. Vascular endothelial and innate immune cells are considered the most relevant cells for FcRn-mediated albumin homeostasis *in vivo.* However, little is known about endocytic trafficking of FcRn–albumin complexes in primary human endothelial cells. To investigate FcRn–albumin trafficking in physiologically relevant endothelial cells, we generated primary human vascular endothelial cell lines from blood endothelial precursors, known as blood outgrowth endothelial cells (BOECs). We mapped the endosomal system in BOECs and showed that BOECs efficiently internalise fluorescently labelled HSA predominantly by fluid-phase macropinocytosis. Pulse-chase studies revealed that intracellular HSA molecules co-localised with FcRn in acidic endosomal structures and that the wildtype HSA, but not the non-FcRn-binding HSA^H464Q^ mutant, was excluded from late endosomes and/or lysosomes. Live imaging revealed that HSA is partitioned into FcRn-positive tubules derived from maturing macropinosomes, which are then transported towards the plasma membrane. These findings identify the FcRn–albumin trafficking pathway in primary vascular endothelial cells, relevant to albumin homeostasis.

## Teaser

**Highlighted Article:** Primary vascular endothelial cells internalise albumin by macropinocytosis, and albumin is then recycled from maturing macropinosomes by an FcRn-mediated process.

## INTRODUCTION

The neonatal Fc receptor (FcRn, also known as FCGRT) is important in both fetal and adult life as it mediates transport of maternal antibodies to the fetal circulation and subsequently is responsible for extension of the serum half-life of immunoglobulin G (IgG) and albumin (Roopenian and Akilesh, 2007; Ward and Ober, 2009). FcRn rescues IgG and albumin, which have been endocytosed by cells, from lysosomal degradation by recycling these ligands from acidic endosomes back to the cell surface (Roopenian and Akilesh, 2007; Ward and Ober, 2009). FcRn is a membrane-bound heterodimer and consists of a ∼40 kDa major histocompatibility complex (MHC) class I-like heavy chain and a non-covalently associated light chain, β2 microglobulin (B2M) (Burmeister et al., 1994). Although related to classical MHC class I molecules, FcRn does not bind peptide antigens; rather, its ligands, IgG and albumin, have independent binding sites that involve histidine residues and pH-dependent salt bridges, which accounts for the ability of FcRn to bind ligands within an acidic endosomal environment, but not at physiological pH at the cell surface (Andersen et al., 2012).

Identification of the pathways for uptake of FcRn ligands and the intracellular trafficking pathways that contribute to this process is important in defining the cell biology of the FcRn recycling system (Roopenian and Akilesh, 2007; Ward and Ober, 2009) and also to enhance the ability to exploit the FcRn pathway by extending the half-life of recombinant proteins for therapeutic applications (Martins et al., 2016). The majority of studies to date on the recycling of FcRn ligands have used immortalised cell lines (Pyzik et al., 2019). As specialised cells show differences in efficiencies of various endocytosis pathways, particularly fluid-phase endocytosis (Lin et al., 2020), and intracellular membrane trafficking pathways, it is important to analyse recycling pathways in primary cells (Anderson, 2014). Of relevance to fluid-phase uptake of FcRn ligands, macropinocytosis is often inefficient in immortalised cells compared with its efficiency in primary cells (Lin et al., 2020).

Endothelial and immune cells are considered the most important tissue and cells *in vivo* for the recycling of FcRn ligands (Montoyo et al., 2009). Of the immune cells, macrophages are considered the most important for FcRn-mediated ligand recycling (Akilesh et al., 2007; Montoyo et al., 2009; Challa et al., 2019). Our previous studies analysed the internalisation and trafficking of human serum albumin (HSA) in genetically modified mouse bone marrow-derived macrophages (BMDMs) that express human FcRn rather than mouse FcRn (Pannek et al., 2022; Toh et al., 2019). We showed that primary macrophages internalise HSA by macropinocytosis, a pathway for the uptake of large volumes of extracellular fluid (Toh et al., 2019). Following endocytosis and interaction with FcRn, albumin was rapidly recycled in tubular transport carriers derived from newly formed macropinosomes (Pannek et al., 2022; Toh et al., 2019).

In contrast to macrophages, there is little information on the recycling of FcRn ligands by primary endothelial cells. Endothelial cells have a large contact area with blood and are known to efficiently endocytose serum proteins (John et al., 2003; Pitas et al., 1985; Smedsrød et al., 1984; Vogel et al., 2001). A major challenge has been the absence of a reliable primary vascular endothelial cell model (Potente and Mäkinen, 2017). From studies using immortalised endothelial cells, a variety of FcRn ligand uptake pathways have been reported, including macropinocytosis and the caveolae-mediated endocytosis pathway, and the receptor-mediated uptake of albumin has also been proposed (Tiruppathi et al., 1997; Ward et al., 2003), although the gene for the putative receptor, known as albondin or gp60, has not been identified. Endocytosed IgG molecules were shown to recycle via Rab11-positive tubular carriers in the immortalised human dermal microvascular endothelial cell (HMEC) line (Ward et al., 2005). It is not known whether the trafficking of albumin follows a similar itinerary in primary endothelial cells. A promising approach is the generation of primary human vascular endothelial cell lines from circulating endothelial progenitor cells found in adult peripheral blood (Martin-Ramirez et al., 2012), as they have high proliferative potential (Lin et al., 2002) and give rise to blood outgrowth endothelial cells (BOECs) (Martin-Ramirez et al., 2012).

To define the albumin-recycling pathway in physiologically relevant primary endothelial cells, we generated primary human vascular endothelial cell lines from peripheral blood, characterised the intracellular localisation of FcRn and defined the mechanism of fluid-phase uptake of HSA and the intracellular fate of internalised albumin using a range of imaging approaches. Our findings demonstrate that macropinocytosis is the major pathway of albumin uptake by primary vascular endothelial cells and that albumin is actively sorted from maturing macropinosomes into tubular transport carriers, prior to delivery of the luminal contents of macropinosomes to late endosomes and/or lysosomes.

## RESULTS

### Generation and characterisation of BOECs isolated from human peripheral blood

For the generation of stable BOEC lines, fresh peripheral blood from healthy donors was collected and peripheral blood mononuclear cells (PBMCs) were cultured in endothelial growth factor-containing BOEC generation medium for up to 4 weeks and monitored for the appearance of endothelial-like cell colonies (Fig. S1). To date, only a few protocols for stable human BOEC generation have been described, ranging from generation of single clones in 48-well plates to large-scale BOEC cultures in multi-layered cell factories (Martin-Ramirez et al., 2012; Ormiston et al., 2015; Reinisch et al., 2009). Most of the established BOEC protocols have been designed for disease-associated studies and/or treatments, in which either single clones are required for characterizing endothelial cell-linked disorders or high BOEC numbers are essential to trigger *de novo* vessel formation *in vivo* after adoptive cell transfer (Martin-Ramirez et al., 2012; Ormiston et al., 2015; Reinisch et al., 2009). In contrast, we only required smaller cell numbers. Therefore, we adapted the protocol for the rapid establishment of endothelial cell lines to collect relatively small numbers of cells with high proliferative properties (Fig. S1B). Isolated PBMCs were directly seeded into collagen type I-coated T-25 flasks and cultured in FBS and endothelial growth factor-rich medium as described in the Materials and Methods (Fig. S1B,C). Following culture for more than 2 weeks, dense and rapidly proliferating cell colonies with a cobblestone-like morphology started to propagate in the culture flasks (Fig. S1D). On average, two to three of these colonies were observed per flask and emerged between days 14 and 28 of culture. Cell colonies were then passaged into collagen type I-coated T-25 flasks, and 2-3 days after sub-passaging, the adherent cells were semi-confluent and exhibited endothelial cell-specific cobblestone-like morphology (Fig. S1E).

**Fig. 1.**
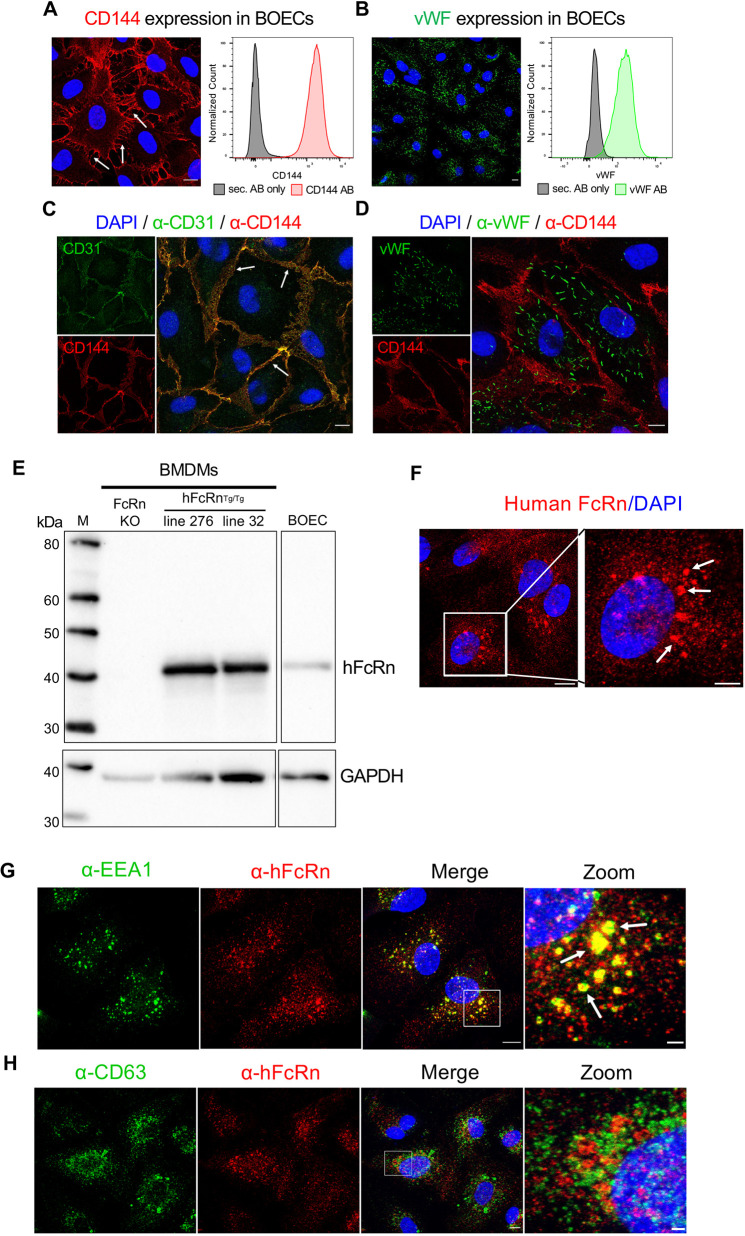
**Expression of endothelial-specific markers and FcRn in established BOEC lines.** (A–D) Confluent monolayers of BOECs were prepared for immunofluorescence microscopy and flow cytometry, as described in the Materials and Methods. (A,B) BOECs were stained with either CD144 (red) (A) or intracellular vWF (green) (B), and shown are confocal images (left) and FACS analysis (right). White arrows (A) indicate CD144 at the PM. Cells were gated on single, live cells and the histograms are shown for CD144-specific (red) and vWF-specific (green) fluorescence signals. Control cells were stained using the respective secondary antibodies only (grey). Scale bars: 10 µm. (C,D) Monolayers of BOECs were fixed with PFA (C) or methanol (D) and either stained for CD31 (green) (C) or vWF (green) (D) and CD144 (red). Cell nuclei were visualised using DAPI. White arrows indicate intracellular junctions between single BOECs. Scale bars: 10 µm. (E) Cultured BOECs and BMDMs isolated from FcRn KO, hFcRn^Tg/Tg^ line 276 or hFcRn^Tg/Tg^ line 32 mice were analysed by immunoblotting, with antibodies against human FcRn or GAPDH after stripping the membrane, and HRP-conjugated secondary antibodies. Chemiluminescence was detected using a ChemiDoc system (Bio-Rad). Data have been consolidated from the same immunoblot. The original uncropped blot is shown in Fig. S7. (F) BOECs were fixed with TCA, permeabilised with 0.1% Triton-X-100 and stained for human FcRn (red), and nuclei were visualised with DAPI (blue). White arrows indicated enlarged FcRn-positive endosomal structures. Scale bars: 10 µm (left); 5 µm (right). (G,H) BOECs were fixed with TCA (G) and methanol (H), and stained for human FcRn (red), either EEA1 (green, G) or CD63 (green, H), and DAPI (blue). In G, white arrows indicate the enlarged FcRn- and EEA1- positive endosomal structures. Scale bars: 10 µm (merge); 2 µm (zoom). Images are representative of more than three independent experiments.

To confirm the endothelial character of established BOEC lines, the expression of vascular endothelial markers was assessed using confocal fluorescence microscopy and flow cytometry (Fig. 1A–D). Vascular endothelial cadherin (VE-cadherin or CD144, encoded by *CDH5*) is a major regulator of endothelial integrity and represents an essential marker for the endothelial phenotype (Harris and Nelson, 2010). The expression of CD144 was verified in the established BOEC lines by confocal fluorescence microscopy and flow cytometry (Fig. 1A). Fluorescence microscopy confirmed the localisation of CD144 to the plasma membrane (PM) and tight junctions between individual BOECs. Furthermore, flow cytometric analyses revealed a single peak of CD144-positive cells, indicating a homologous population of BOECs exhibiting an endothelial phenotype. Established BOEC lines were analysed regularly for CD144 expression during sub-passaging. BOEC lines were >94% positive for CD144 at early passages (e.g. passage 5 and 6), and morphology and growth were similar between different lines. The cell surface levels of CD144 waned gradually with passage number. As an example, one of the BOEC lines was 99.7% CD144-positive at P5, 96.7% at P12 and 90.8% at P14. The experiments described in this study used two different cell lines derived from two different donors, and CD144 levels were >90% for all experiments.

The expression of platelet endothelial cell adhesion molecule (PECAM-1 or CD31) (Watt et al., 1995), located at the intercellular junction of endothelial cells, was also assessed. Co-staining of CD31 and CD144 revealed the co-localisation of both markers to the cell borders of BOECs (Fig. 1C). In addition to the staining for endothelial cell-specific surface markers of BOECs, intracellular von Willebrand factor (vWF) expression was evaluated in BOEC lines (Fig. 1B,D). The glycoprotein vWF is one of the main components of endothelial cell-specific storage organelles, namely, Weibel–Palade bodies (WPBs), which exhibit a characteristic rod-shaped morphology. vWF was expressed in over 95% of BOECs as detected by flow cytometry (Fig. 1B), and microscopy revealed the localisation of vWF to rod-shaped intracellular organelle structures, suggesting the association of vWF with WPBs (Fig. 1D). In endothelial cells, CD63, a marker for late endosomes, is also associated with WPBs (Vischer and Wagner, 1993), and the co-localisation of vWF and CD63 indicated the presence of WPBs in BOECs (Fig. S2A). BOECs were treated with phorbol-12-myristate-13-acetate (PMA), which is known to trigger release of WPBs in primary endothelial cells (Nightingale et al., 2018). BOECs treated with 80 nM PMA for 20 min showed minimal staining for vWF, demonstrating that intracellular vWF was associated with functional endothelial storage granules. Collectively, these observations support the endothelial character of these human primary BOEC lines.

### Characterisation of endosomal compartments in BOECs

To study intracellular trafficking pathways in BOECs, we mapped the distribution of endosomal compartments and their morphology using endosomal markers, as endosome populations have not been previously characterised in BOECs. CD63 and LAMP1 are selective markers for late endosomes and lysosomes, respectively, and these markers were localised to large punctate structures, with the majority of signals in close proximity to the cell nucleus in fixed BOECs (Fig. S3A). There was considerable overlap between CD63 and LAMP1, typical of late endosome and lysosome staining. The spatial relationship of late endosomal structures with the Rab11-positive recycling compartment was also assessed (Fig. S3B). Both CD63 and Rab11 markers were concentrated in the juxtanuclear position; however, there was very little overlap between the two compartments.

There was also no overlap between the cis-Golgi marker GM130 (or GOLGA2) and Rab11, and only minor overlap with a TGN marker, golgin-97 (or GOLGA1), and Rab11 (Fig. S3C,D). The Rab11-positive structures were closely associated with the Golgi apparatus; however, the co-staining data demonstrate that the recycling endosomes and the Golgi can be readily distinguished.

### Expression and intracellular localisation of FcRn in BOECs

To characterise FcRn in BOECs, we analysed the expression of FcRn in the established cell lines. Cell lysates were analysed by western blotting using a polyclonal antibody specific for human FcRn heavy chain (Fig. 1E). A FcRn-specific band of ∼45 kDa was observed in cell lysates of BOECs, confirming the endogenous expression of FcRn in these primary vascular endothelial cells. We also included cell lysates of mouse BMDMs for comparison, which included an FcRn knockout (KO) line and two different humanised mouse BMDM lines. The two humanised mouse models harbour a KO allele of the murine FcRn and express the heavy chain of human FcRn transgenically under the control of the strong chicken β-actin promoter (line 276) or the native human FcRn promoter (line 32) (Chaudhury et al., 2003; Petkova et al., 2006). As expected, a band corresponding to the size of human FcRn α-chain of ∼45 kDa was detected in extracts of murine lines 276 and 32, but not in FcRn KO BMDMs (Fig. 1E). Quantitation of FcRn α-chain from replicate immunoblots showed that the levels in BOECs were approximately 15% of the levels in line 276 and 40% of the levels of line 32 (Fig. S2B). The difference in FcRn expression between line 32 BMDMs, in which the transgene is under the control of the endogenous FcRn promoter, and BOECs is consistent with human single-cell RNA-sequencing data, in which the expression of FcRn in macrophages has been reported to be more than twice that of endothelial cells (The Human Protein Atlas, 2021; https://www.proteinatlas.org/ENSG00000104870-FCGRT/celltype; Uhlén et al., 2015). Hence, FcRn expression levels in BOECs are compatible with the expected levels in the primary vascular endothelium.

To determine the intracellular localisation of FcRn in BOECs, fixed and permeabilised BOECs were stained using a polyclonal antibody against human FcRn (Fig. 1F). FcRn was associated with endosome-like structures throughout the cytoplasm and, notably, was localised predominantly to enlarged endosomal structures with diameters up to 2 µm (indicated by white arrows). The association of FcRn with large endosomal structures was observed using either Triton X-100- (Fig. 1F) or saponin-based permeabilisation protocols (not shown), which indicates that these large structures are not an artefact of the permeabilisation method.

BOECs were co-stained for FcRn and early or late endosomal markers, EEA1 and CD63 (Fig. 1G,H), respectively, to determine the distribution of FcRn in the endo-lysosomal pathway. FcRn-positive structures co-localised strongly with EEA1 (Fig. 1G). There was heterogeneity of the size of FcRn- and EEA1-positive structures, with some structures having diameters up to 2 µm, suggesting the presence of FcRn not only in early endosomes but also in early macropinosomes. Co-staining with CD63 revealed no detectable overlap with FcRn, indicating the absence of the receptor in late endosomal and lysosomal compartments (Fig. 1H).

### Introduction of FcRn–mCherry in BOEC lines

As the expression of a fluorescently tagged FcRn would allow the direct observation of FcRn in live BOECs, we established expression of mCherry-labelled human FcRn in cultured BOECs. Transient transfection using Lipofectamine 3000 gave only a low level of transfection; hence, lentiviral transduction of BOEC was used. The open reading frames encoding β2 microglobulin and FcRn–mCherry from an internal ribosome entry site (IRES)-containing bicistronic vector (Pannek et al., 2022) were introduced into the plasmid backbone pFUGW under the control of the constitutive UbC promoter, which drives low ectopic gene expression in most mammalian cells (Qin et al., 2010). Lentiviral particles containing the pFUGW-B2M-FcRn-mCherry plasmid were generated and BOECs were transduced with recombinant lentivirus. Immunoblotting detected a ∼72 kDa band in transduced cells that corresponded to the predicted size of the FcRn–mCherry fusion protein and a 45 kDa band corresponding to endogenous FcRn α-chain in both non-transduced and transduced cells; a ∼72 kDa component was also recognised by an anti-mCherry antibody, confirming the identity of the ∼72 kDa band as FcRn–mCherry (Fig. S4A). The lower, weaker band of ∼30 kDa detected by anti-mCherry corresponds to the size of free mCherry and is likely due to the cleavage of mCherry from the fusion protein either within the cell or upon cell extraction; this soluble mCherry fragment would be diffusely distributed in the cytosol and would represent a minor fluorescence signal. Based on the immunoblot, the FcRn pool in transduced BOECs consisted of approximately one-third the transgenically introduced FcRn–mCherry and two-thirds the endogenous FcRn, and provided a strategy for direct observation of FcRn–mCherry localisation in live cells.

Transduced BOECs showed >90% cells positive for FcRn–mCherry fluorescence, indicating a very high transduction efficiency (Fig. S4B). FcRn–mCherry was localised to endosomal structures throughout the cell periphery reaching diameters up to 2 µm (Fig. S4B, indicated by white arrows).

### Dynamic FcRn trafficking in live BOECs

**Fig. 2.**
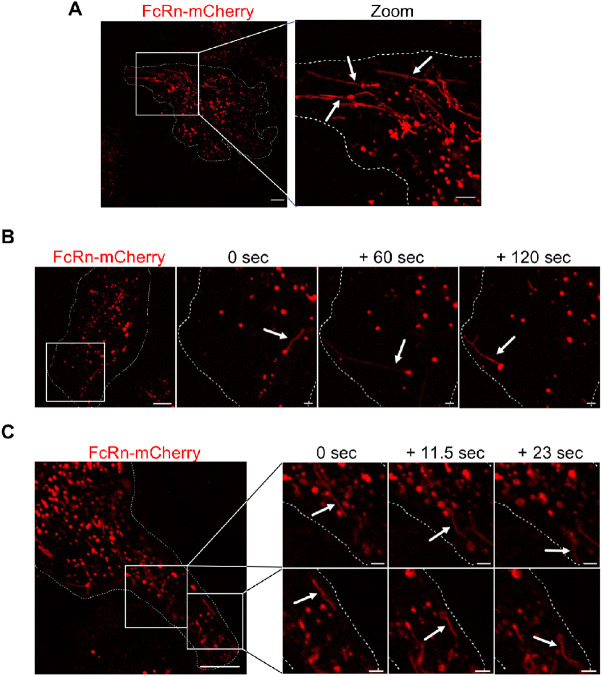
**Dynamic trafficking of FcRn–mCherry in transduced live BOECs.** BOECs were transduced with 10 µl lentivirus containing pFUGW-B2M-FcRn_mCherry (red) for 24 h, the monolayers were washed and cells were incubated for an additional 24 h. Live BOECs were imaged at 37°C and 5% CO_2_ with a FV3000 Olympus confocal microscope. (A) Representative image showing the presence of tubular structures. White arrows indicate FcRn–mCherry-positive tubules emerging from endosomal structures. Cell boundaries are indicated by dotted lines. Scale bars: 10 µm (FcRn–mCherry); 5 µm (zoom). (B,C) Time lapse of live cells was recorded either with a pause of 60 s (B) or continuously (frame speed=5.84 s) (C). Representative frames are shown. Cell boundaries are indicated by dotted lines. White arrows indicate tubular structures. Scale bars: 10 µm (left); 2 µm (zoomed frames). Images are representative of more than three independent experiments.

To visualise the dynamics of intracellular FcRn trafficking in BOECs, live microscopy using FcRn–mCherry-transduced cells was carried out, and an image of a cell is shown in Fig. 2A. The FcRn–mCherry fusion protein was predominantly localised to large globular structures throughout the cytoplasm of transduced cells. Many of these structures had a diameter of 1–2 µm, suggesting the association of FcRn–mCherry with macropinosomes. Interestingly, FcRn–mCherry was not only localised to endosomal puncta but also to tubular structures, corresponding to the size of tubular transport carriers, emerging from these large globular structures (Fig. 2A, indicated by white arrows). These tubular carriers were up to 20 µm long, ∼450 nm in diameter and appeared to be directed towards the edges of the cell. The majority of the tubular carriers had a length of around 2–5 µm. To further assess the dynamics and direction of these FcRn–mCherry-positive tubular transport carriers, a time series of single transduced BOECs was recorded and single tubular events followed over a 120 s period (Fig. 2B). A tubule can be observed arising from an endosome, which then extends and approaches the PM. The transit of FcRn–mCherry-positive tubular transport carriers was also analysed within shorter time intervals during a continuous acquisition timeframe (Fig. 2C; Movie 1). Highly dynamic movement of these tubular transport carriers directed to the outer perimeter of the cell was observed. Within a 23 s observation timeframe, the FcRn–mCherry-positive tubules moved about 10 µm within the cytoplasm of transduced BOECs, corresponding to a velocity of approximately 0.43 µm/s.

Taken together, analysis of FcRn–mCherry in BOECs by live imaging revealed a tubulovesicular endosomal network facilitating highly dynamic FcRn trafficking in primary vascular endothelial cells, and FcRn–mCherry-positive tubular transport carriers emerging from enlarged globular endosomes were observed moving towards the edges of the cell.

### FcRn–mCherry does not localise to Rab11-positive compartments in live BOECs

**Fig. 3.**
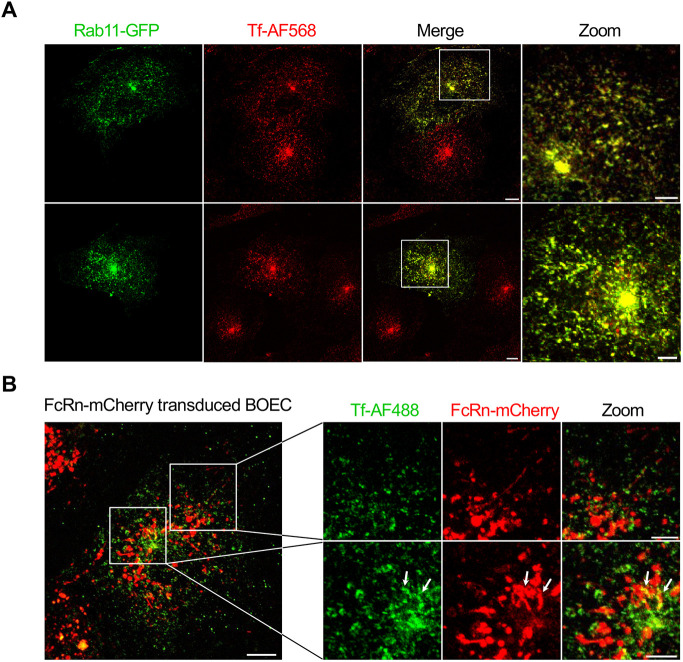
**FcRn–mCherry does not reside in the recycling endosomes in transduced live BOECs.** (A) BOECs were transfected with Rab11–GFP (green) using Lipofectamine 3000. After 48 h, cells were pulsed for 15 min with Alexa Fluor 568-labelled transferrin (Tf–AF568, red) at 37°C. (B) BOECs were transduced with 10 µl lentivirus containing pFUGW-B2M-FcRn_mCherry (red) for 24 h, and the monolayers were washed and incubated for an additional 24 h. Transduced cells were pulsed for 15 min with Tf–AF488 (green) at 37°C. (A,B) Monolayers of BOECs were washed and live cells imaged at 37°C and 5% CO_2_ with a FV3000 Olympus confocal microscope. White arrows indicate low level of overlap in the perinuclear region. Images represent maximum projections of whole-cell *z*-stacks from two independent experiments. Scale bars: 10 µm (original images); 5 µm (zoomed images).

As described above, endogenous FcRn–mCherry in BOECs was predominantly localised to EEA1-positive but not to CD63-positive endosomes. In a number of immortalised cells including immortalised endothelial cells, the recycling of FcRn occurs via Rab11^+^ recycling endosomes (Chia et al., 2018; Ward et al., 2005); however, whether FcRn transits the recycling endosome in primary endothelial cells is not known. The association of FcRn with Rab11-positive recycling endosomes in BOECs could not be evaluated in fixed FcRn–mCherry-expressing cells owing to an incompatibility of the fixation conditions required for Rab11 staining and the preservation of mCherry fluorescence or human FcRn staining. Therefore, we introduced Rab11–GFP in BOECs to allow direct observation of Rab11-positive recycling endosomes (Fig. 3). Fluorescently labelled transferrin (Tf), which is known to transit recycling endosomes after internalisation, showed extensive co-localisation with Rab11–GFP in transfected BOECs, confirming that both molecules were present in recycling endosomes (Fig. 3A). To assess whether FcRn was localised to recycling endosomes, FcRn–mCherry-transduced BOECs were pulsed with Alexa Fluor 488 (AF488)-conjugated transferrin (Tf–AF488) for 15 min and the localisation of both molecules assessed (Fig. 3B). FcRn–mCherry and Tf–AF488 were associated with distinct endosomal structures, no co-localisation was observed in the cell periphery and only a low level of overlap was observed between FcRn- and transferrin-positive structures in the juxtanuclear region (Fig. 3B, indicated by white arrows). These data strongly indicate that FcRn does not reside or transit the recycling endosomes of BOECs.

### Uptake of HSA by BOECs occurs by macropinocytosis

**Fig. 4.**
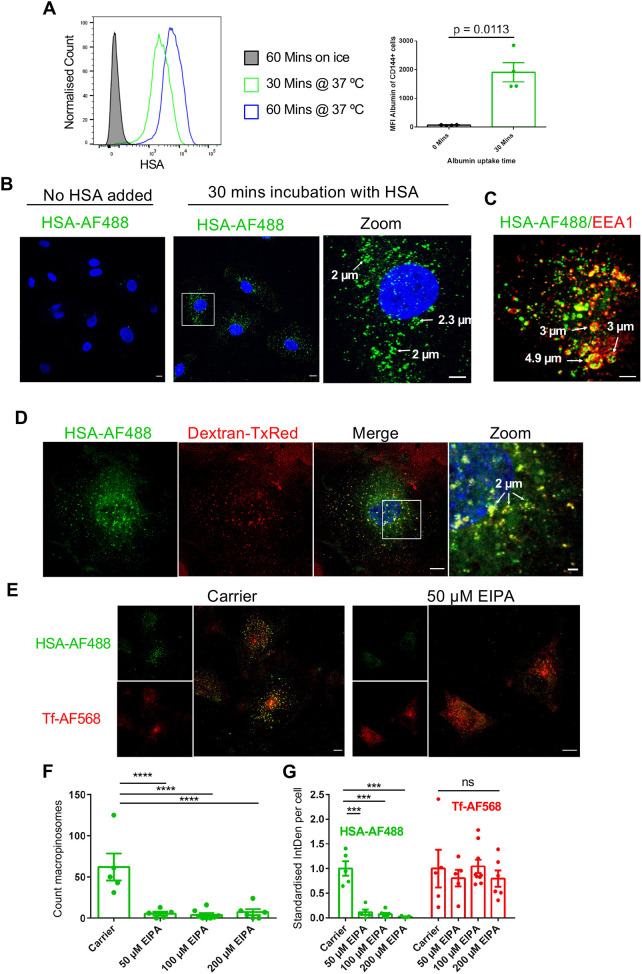
**HSA uptake in cultured BOECs.** (A) Cultured BOECs were pulsed for 30 min (green) or 60 min (blue) with HSA–AF568 at 37°C or on ice (grey). Monolayers were washed, cells detached from culture vessels and stained for CD144, and dead cells identified by DAPI staining. HSA uptake was analysed by flow cytometry. The mean fluorescence intensity (MFI) was calculated to quantify HSA uptake and data for 30 min uptake were pooled from four individual experiments. Data were analysed using a two-tailed paired Student's *t*-test and error bars represent s.e.m. (B,C) BOECs were pulsed for 30 min with HSA–AF488 (green) at 37°C. Monolayers were fixed and HSA–AF488 was uptake examined directly (B) or after staining for EEA1 (red) (C). Nuclei were visualised using DAPI (blue). Enlarged HSA-positive structures are highlighted by white arrows and the estimated diameter is shown. Images represent maximum projections of *z*-stacks from more than three independent experiments. Scale bars: 10 µm (original images); 5 µm (zoom). (D) BOECs were pulsed with HSA–AF488 (green) and 70 kDa dextran labelled with TexasRed (Dextran-TxRed) for 30 min at 37°C. After internalisation, cells were fixed with PFA and imaged by confocal fluorescence microscopy. Nuclei were visualized using DAPI. Shown is the maximum-intensity projection of *z*-stack optical sections. Enlarged HSA- and dextran-positive endosomal structures are indicated by white arrows and their estimated diameter is shown. Images are representative of three independent experiments. Scale bars: 10 µm; 2 µm (zoom). (E–G) BOECs were pre-treated with EIPA or with methanol alone (carrier) for 60 min at 37°C, before performing uptake experiments in the presence of the indicated EIPA concentration. EIPA-treated BOECs were pulsed with HSA–AF488 (green) and Tf–AF568 (red) at 37°C for 30 min. (E) Cells were fixed and imaged by confocal microscopy. Scale bars: 10 µm. (F) HSA–AF488-positive structures (macropinosomes; ∼0.5–5 µm diameter) from *z*-stacks of fixed cells were quantified. (G) Intracellular fluorescence levels (integrated density, IntDen) per cell for HSA–AF488 and Tf–AF568 were quantified using *z*-stack projections. Integrated densities were normalised to the mean values of the carrier controls (F). Data were analysed using Fisher's LSD test. Error bars represent s.e.m. ns, not significant; ***P<0.001; ****P<0.0001.

A variety of different mechanisms have been claimed to be responsible for the uptake of the albumin by immortalised endothelial cells, including macropinocytosis, receptor-mediated endocytosis involving the albumin receptor albondin or gp60 (Tiruppathi et al., 1997; Ward et al., 2003), and the caveolae-mediated endocytosis pathway (Li et al., 2013). To evaluate how HSA uptake is facilitated in primary vascular endothelial cells, BOECs were incubated with fluorescently labelled HSA (HSA–AF488) and HSA endocytosis was analysed by flow cytometry and confocal fluorescence microscopy (Fig. 4). Flow cytometric analysis of BOECs pulsed with HSA–AF488 at 37°C revealed a strong HSA signal after 30 min (mean fluorescence intensity, 1.9×10^3^) and 60 min (mean fluorescence intensity, 7.3×10^3^) uptake. These results were consistent across several experiments (*n*=4) (Fig. 4A). Incubation of BOECs with HSA–AF488 on ice for 60 min showed no detectable fluorescence, indicating that there was no binding of HSA–AF488 to the cell surface of BOECs (Fig. 4A). To evaluate the endocytosed HSA in BOECs, cells were pulsed with HSA–AF488 for 30 min, fixed with paraformaldehyde (PFA) and assessed by confocal microscopy (Fig. 4B). HSA fluorescence was associated with enlarged donut-shaped endosomal structures. Co-staining with the early endosomal and macropinosomal marker EEA1 in HSA-pulsed BOECs identified the presence of large HSA- and EEA1-double-positive endosomal structures in BOECs (Fig. 4C). In some cells, these enlarged donut-shaped structures, defined by EEA1 on the limiting membrane, were observed to have a size of up to 4.9 µm in diameter (Fig. 4C, indicated by white arrows). See also live imaging of HSA–AF488 chase in BOECs (Movie 2).

We further investigated macropinocytosis activity in BOECs. 70 kDa-dextran is widely used as a marker for fluid-phase macropinocytosis (Li et al., 2015), and a co-uptake of HSA–AF488 and 70 kDa-dextran was performed to confirm the macropinocytic activity of BOECs (Fig. 4D). Endocytosed HSA and 70 kDa-dextran co-localised in large endosomal structures with diameters up to 2 µm. A Pearson's correlation coefficient of 0.83 was calculated for the overlap of the fluorescence signals corresponding to HSA and 70 kDa-dextran.

Macropinocytosis can be inhibited in cultured cells by treatment with the amiloride derivative 5-(N-ethyl-N-isopropyl)amiloride (EIPA). Treatment of BOECs with 50 µM EIPA led to a significant reduction of HSA uptake in BOECs compared with that in the carrier control (Fig. 4F). Macropinosome-like structures with a diameter of 0.5–5 µm were quantified using an ImageJ-based macropinosome analysis adapted from Wang et al. (2014) (Fig. 4E,F). In the carrier control, on average, 62 HSA-positive macropinosome-like structures were detected per cell, whereas the treatment with 50 µM EIPA dramatically reduced the presence of HSA-positive macropinosome-like structures to 5.5 per cell. The treatment of BOECs with higher concentrations of EIPA (100 and 200 µM) showed a similar reduction in macropinosome number (Fig. 4F). Total HSA–AF488 fluorescence levels per cell were also calculated by using *z*-projections and appropriate thresholding algorithms, and the results confirmed that the uptake of HSA was strongly inhibited by EIPA treatment (Fig. 4G). In contrast to HSA, the uptake of transferrin in BOECs was not impaired by the presence of EIPA (Fig. 4G), indicating that receptor-mediated endocytosis was not affected by EIPA. These data demonstrate that HSA is predominantly taken up into BOECs by macropinocytosis.

### Fate of endocytosed HSA in BOECs

**Fig. 5.**
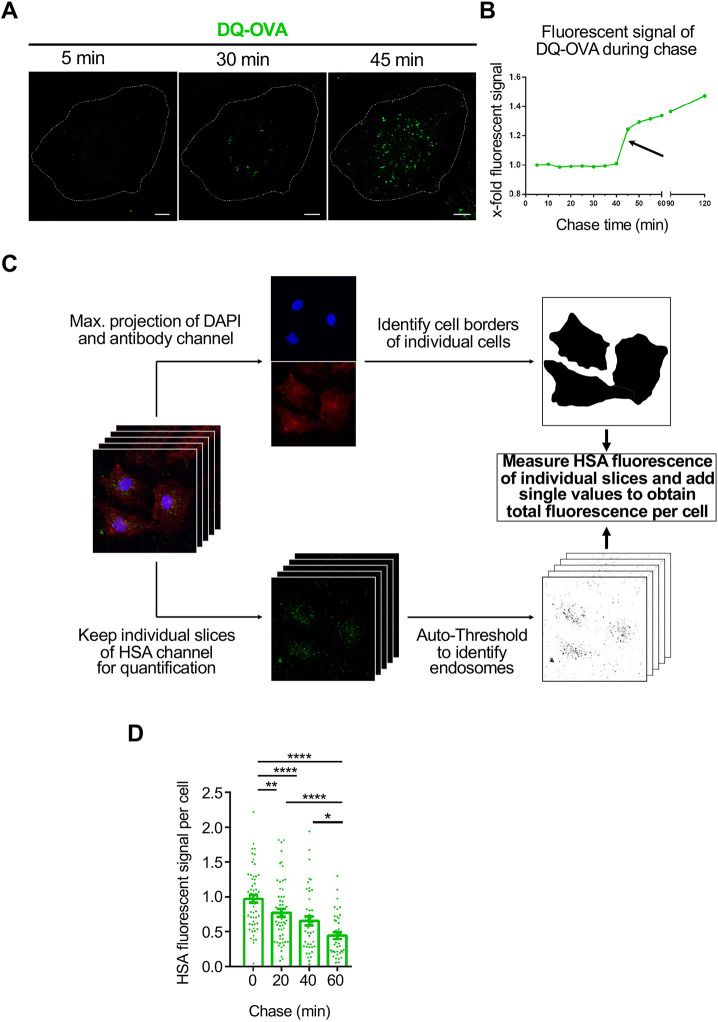
**Pulse-chase analysis of DQ-OVA and HSA in BOECs.** (A,B) Pulse-chase analysis of DQ-OVA in BOECs. (A) BOECs were pulsed with DQ-OVA (green) for 15 min at 37°C and DQ-OVA fluorescence was assessed over a 120 min chase in live cells. Cells were imaged every 5 min at 37°C using an Olympus FV3000 confocal fluorescence microscope. Cell boundary is indicated by dotted lines. Images are representative of two independent experiments. Scale bars: 10 µm. (B) The DQ-OVA fluorescence in confocal images of the live BOEC from A was quantified over the chase time. The total cell fluorescence intensity at 0 min (immediately after DQ-OVA uptake) was set to 1 and values expressed as fold change (x-fold). The black arrow indicates the appearance of bright DQ-OVA fluorescence after 45 min of chase. (C,D) Disappearance of intracellular HSA fluorescence during pulse-chase experiments in fixed BOECs. BOECs were pulsed with HSA–AF488 for 30 min and the fluorescence signal chased at 37°C for the designated interval. (C) Schematic overview of the protocol for the quantification of intracellular HSA fluorescence in fixed BOECs. Maximum projections of acquired *z*-stacks were made for the DAPI and an unrelated antibody channel to create masks representing the outline of single cells. (D) Quantitation of the total intracellular HSA fluorescence per cell in fixed BOECs. *n*≥19 cells from five independent experiments. Data were analysed using Fisher's LSD test. Error bars represent s.e.m. *P<0.05; **P<0.01; ****P<0.0001.

To investigate the itinerary of HSA transport in cultured BOECs, we initially used the reporter ovalbumin-derived conjugate DQ-OVA, known to be transported directly into the endolysosomal system, to evaluate the time required following fluid-phase uptake of macromolecules to reach the lysosomal compartment in BOECs. (Fig. 5). DQ-OVA is a fluorescent probe extensively labelled with BODIPY FL fluorophores, and in its native state, it does not fluoresce owing to self-quenching. However, upon proteolytic degradation of DQ-OVA, the BODIPY fluorophores released from the DQ-OVA polypeptide exhibit bright fluorescence, indicating processing of the fluorescent DQ-OVA probe in the late endosomes and/or lysosomes. BOECs were pulsed with DQ-OVA for 15 min and the DQ-OVA fluorescence signal was monitored over 120 min (Fig. 5A,B). Minimal fluorescence was observed for the first 40 min of the chase period; after 45 min of chase, bright DQ-OVA fluorescence was observed in live BOECs, indicating proteolytic degradation of the endocytosed ovalbumin conjugate (Fig. 5A,B). Quantification of the DQ-OVA fluorescence showed a sharp increase of DQ-OVA at the 45-min timepoint, indicating that endocytosed DQ-OVA cargo had reached degradative lysosomal compartments at the 45-min timepoint and, subsequently, DQ-OVA fluorescence rose steadily as degradation of the DQ-OVA conjugates proceeded.

**Fig. 6.**
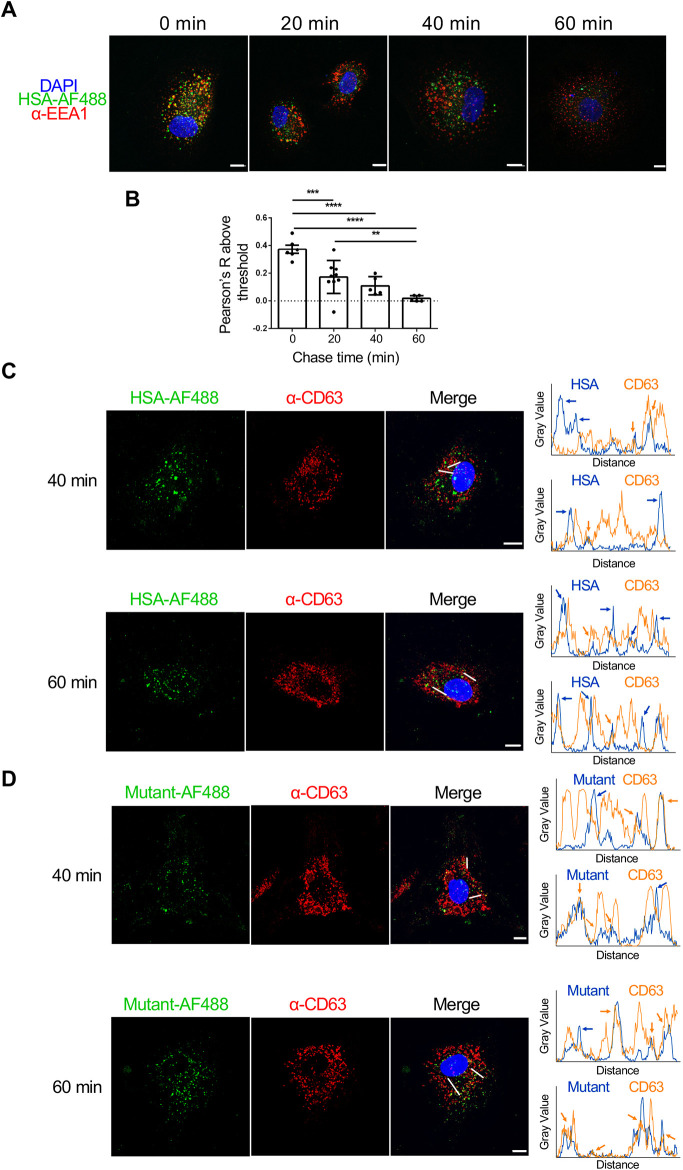
**Pulse-chase of internalised HSA and HSA^H464Q^ with endosomal and/or lysosomal markers in fixed BOECs.** (A–D) BOECs were pulsed with either HSA–AF488 (green) (A–C) or non-FcRn-binding HSA^H464Q^ (Mutant–AF488, green) (D) for 30 min at 37°C. Monolayers were washed and the intracellular fluorescence signal chased for up to 60 min at 37°C. After the chase periods, cells were directly fixed with PFA and stained for EEA1 (red; A,B) or CD63 (red; C,D). Nuclei were visualised using DAPI (blue). Images represent maximum projections of whole-cell *z*-stacks from more than three independent experiments. Scale bars: 10 µm. (B) Quantification of co-localisation by Manders' correlation coefficient M1. The parameters were calculated using ImageJ and values shown for calculated parameters above threshold; *n*≥5 cells from one experiment. Data were analysed using Fisher's LSD test. Error bars represent s.e.m. **P<0.01; ***P<0.001; ****P<0.0001. (C,D) The profiles of two line scans per image are shown for both fluorophores. The location of the respective line scans is indicated by white lines. Images represent maximum projections of whole-cell *z*-stacks. Data are representative of more than three independent experiments. Scale bars: 10 µm.

The disappearance of the internalised HSA fluorescence signal was evaluated by pulse-chase experiments. Cultured BOECs were pulsed for 30 min with HSA–AF488 to accumulate sufficient labels for analysis, and the internalised signal was then quantified within individual cells from three-dimensional images using the protocol outlined in Fig. 5C. The fluorescence signal gradually decreased over the 60 min chase period and, at this time point, only low levels of HSA fluorescence could be detected by microscopy (Fig. 6A). The total HSA fluorescence per cell was normalised to the value for 0 min chase and the data obtained from five independent pulse-chase experiments were pooled to evaluate the disappearance of endocytosed HSA in fixed BOECs (Fig. 5D). During the pulse chase, the total HSA fluorescence level per cell dropped gradually from 100% from the beginning of the chase to ∼45% after a 60-min chase (Fig. 5D). In particular, the reduction in HSA fluorescence per cell during the first 40 min of chase suggests that recycling might be responsible for the loss of fluorescence, as this time period was insufficient for HSA to be transported to late endosomes and/or lysosomes, based on DQ-OVA uptake analysis.

### Intracellular trafficking of HSA in BOECs

The intracellular itinerary of endocytosed HSA was investigated in fixed BOECs by co-staining with endosomal markers. After a 30 min uptake of HSA–AF488 by BOECs, there was strong co-localisation with EEA1, a marker of both early endosomal and macropinosome compartments (Fig. 6A). With increasing chase time, the co-localisation of HSA and EEA1 steadily decreased over 40 min, and by 60 min chase, no overlap was observed (Fig. 6A). Quantitation of the fluorescence overlap is shown in Fig. 6B.

Next, the overlap of HSA with the late endosomal and lysosomal markers, CD63 (Fig. 6C) and LAMP1 (Fig. S5A), respectively, was investigated during the chase. After either 40 or 60 min of chase, there was only a low level of co-localisation of HSA- and CD63-positive endosomal structures in fixed BOECs (Fig. 6C). Line scans revealed that most fluorescent peaks of both molecules did not overlap, rather, they were offset (indicated by blue arrows). Only a minor fraction of HSA–AF488 and CD63 fluorescence peaks overlapped (indicated by orange arrows), indicating a low level of co-localisation between HSA–AF488 and CD63.

We also assessed the overlap of the non-FcRn-binding HSA variant HSA^H464Q^ (indicated as ‘Mutant’) with endo-lysosomal markers following 40 and 60 min chase periods. An overlap of HSA^H464Q^–AF488 and CD63-positive endosomal structures was observed (Fig. 6D). In particular, after 60 min chase, most of the fluorescence peaks for both molecules showed overlap, indicating the association of the non-FcRn-binding HSA mutant with CD63-positive structures (indicated by orange arrows on line scans). Only a small fraction of HSA^H464Q^–AF488 fluorescence peaks did not overlap with CD63 signals (indicated by blue arrows).

Similar trends were observed for the co-localisation of HSA with LAMP1-positive compartments (Fig. S5). HSA did not overlap with the respective peaks of LAMP1 fluorescence after either 40 or 60 min of chase (Fig. S5A), whereas extensive overlap was observed for the non-binding mutant HSA^H464Q^ and LAMP1 (Fig. S5B). Taken together, these data indicate that endocytosed HSA–AF488 is largely excluded from late endosomal and lysosomal structures in fixed BOECs, whereas the non-FcRn-binding HSA variant HSA^H464Q^ was trafficked to late endosomal and lysosomes, suggesting a role for FcRn in regulating the sorting of internalised HSA within the endosomal-lysosomal pathway in BOECs.

### Intracellular trafficking of HSA to acidic, FcRn-positive endosomes in live BOECs

**Fig. 7.**
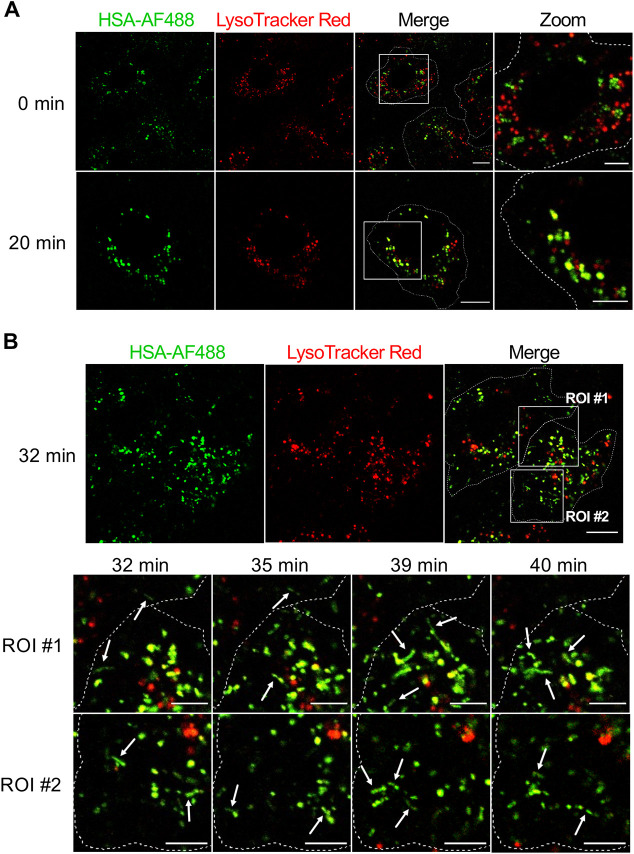
**Localisation of endocytosed HSA–AF488 to LysoTracker Red-positive compartments in live BOECs.** Cultured BOECs were stained with LysoTracker Red DND-99 (red) for 60 min at 37°C and subsequently pulsed with HSA–AF488 (green) for 15 min at 37°C. Monolayers were washed and the HSA–AF488 fluorescence signal was chased for 40 min in live cells. (A) Example images at 0 and 20 min chase. (B) Image of a LysoTracker Red-stained BOEC pulsed with HSA–AF488 after 32 min chase. Two ROIs were defined to highlight the presence of HSA–AF488-positive tubular carriers (indicated by white arrows). Frames of the two ROIs are shown for the 32–40 min chase. Live cells were imaged at 37°C and 5% CO_2_ using an Olympus FV3000 confocal fluorescence microscope. Cell boundaries are indicated by dotted lines. Images are representative of cells from one experiment. Scale bars: 10 µm (original images) and 5 µm (zoomed images).

To decipher the intracellular itinerary of HSA in BOECs, live imaging of a HSA–AF488 pulse-chase experiment was performed. As acidic compartments are required for interaction of HSA with FcRn, we used LysoTracker Red to mark acidic endosomal structures in live BOECs during the pulse-chase assay to determine the kinetics of the arrival of HSA–AF488 within acidic compartments. Upon endocytosis, HSA–AF488 fluorescence was detected in punctate structures in the cell periphery. These structures were negative for LysoTracker Red, indicating a neutral luminal pH of HSA-positive endosomes and/or macropinosomes (Fig. 7A). After 20 min chase, the majority of HSA–AF488 fluorescence overlapped with LysoTracker Red-positive compartments (Fig. 7A), indicating the acidification of HSA-positive macropinosomes, which is a compatible environment for the interaction of endosomal HSA with FcRn. With increasing chase time, the co-localisation of HSA–AF488 and LysoTracker Red fluorescence increased and, after 32 min of chase, tubular carriers were detected pinching off from globular macropinosomes (Fig. 7B, indicated by white arrows; Movie 2). HSA–AF488-positive tubular carriers continued to emerge from LysoTracker Red-positive globular structures with increased chase time (up to 40 min) and were directed to the cell periphery (Fig. 7B).

**Fig. 8.**
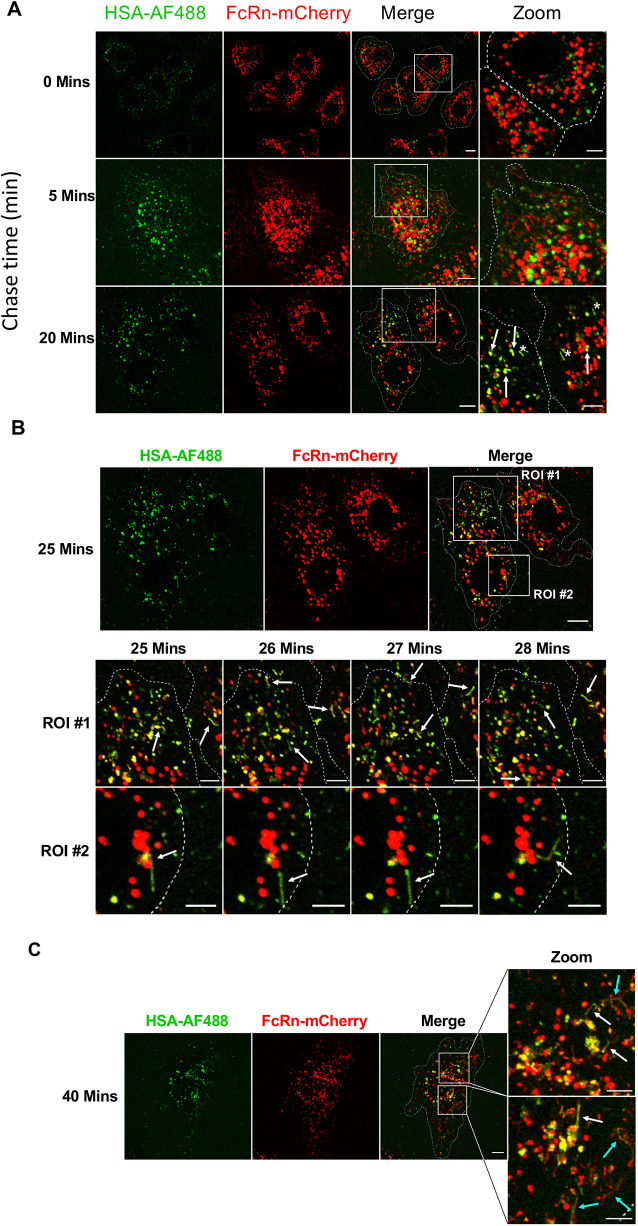
**Intracellular trafficking of HSA–AF488 in FcRn–mCherry-transduced live BOECs.** (A–C) BOECs transduced with FcRn–mCherry (red) were pulsed with HSA–AF488 (green) for 15 min at 37°C and the fluorescence signal was chased for 40 min in live cells. Confocal microscopy images of live cells were taken immediately after the pulse (0 min) (A) or at 5, 20 (A), 25 (B) or 40 (C) min chase. In B, the field from 20 min in A was monitored for the 25–28 min chase time, and zoomed images of ROIs are shown. In C, shown is the maximum-intensity projection of *z*-stack optical sections. White arrows indicate HSA–AF488- and FcRn–mCherry-double-positive endosomal structures. White asterisks (A) indicate HSA–AF488-positive tubular transport carriers. Sky blue arrows (C) indicate tubular carriers that were negative or only faintly positive for HSA–AF488. Cell boundaries are indicated by dotted lines. Images are representative of more than three independent experiments. Scale bars: 10 µm (merge); 5 µm (zoom).

To evaluate the co-localisation of HSA and FcRn within endosomal structures in live cells, BOECs were transduced with FcRn–mCherry and pulsed with HSA–AF488 for 15 min at 37°C, and internalised HSA–AF488 was then chased for 40 min (Fig. 8). The shorter pulse time of 15 min could be used in live-cell experiments as the intracellular fluorescence signal was more intense in the absence of cell fixation. A chase time of 40 min was chosen as this was the chase time prior to arrival in the late endosome and/or lysosomal compartment, as defined above using DQ-OVA. After a 5 min chase time, HSA–AF488 fluorescence was detected in globular macropinosomes in the cell periphery, which were negative for FcRn–mCherry, indicating that FcRn is not localised to newly formed macropinosomes in BOECs (Fig. 8). By 20 min chase, substantial overlap of HSA–AF488- and FcRn–mCherry-specific fluorescence signals was detected, indicating co-localisation of HSA and FcRn in globular macropinosome structures (Fig. 8A; Movie 3). In addition to the globular structures, HSA–AF488 tubular structures were detected after 20 min chase (indicated by white asterisks). With ongoing chase time, tubular structures positive for HSA–AF488 and also FcRn–mCherry were observed more frequently (Fig. 8B; Movie 3). To examine whether these tubular carriers are directed to the cell periphery, several frames of regions of interest (ROIs) in an interval of 1 min were recorded between 25 and 28 min of chase time (Fig. 8B). The inspection of single frames revealed that HSA–AF488-positive tubular carriers (indicated by white arrows) emerged directly from HSA–AF488- and FcRn–mCherry-double-positive maturing macropinosome structures and were directed to the cell surface, rather than being trafficked to the perinuclear region of the cell (Fig. 8B).

The emergence of HSA–AF488- and FcRn–mCherry-positive tubular transport carriers was also detected at the 40 min chase time (Fig. 8C). To characterise the dimensions of the transport carriers, *z*-stacks of whole live BOECs were recorded and displayed as maximum projections (Fig. 8C). This allowed the tubular transport carriers, usually emerging along the *z*-axis, to be followed more readily. Investigation of projected whole-cell stacks revealed the existence of tubular transport carriers positive for HSA–AF488 and FcRn–mCherry (indicated by white arrows) and also FcRn-positive tubular carriers that were negative or only faintly positive for HSA–AF488 (indicated by sky blue arrows, Fig. 8C), indicating that, at this time point, there was unoccupied FcRn. These tubular carriers were 0.5–0.6 µm in diameter and reached lengths up to 7 µm.

Collectively, these data revealed that directly upon endocytosis, AF488-labelled HSA is initially localised to globular macropinosomes devoid of FcRn–mCherry. HSA was observed to co-localise with FcRn–mCherry after 10 min chase, and with increasing chase time, HSA–AF488-positive transport carriers emerged from globular double-positive structures. These transport carriers were partially positive for FcRn–mCherry, suggesting the mutual sorting of FcRn–mCherry and its ligand, HSA–AF488, into these structures. The absence of FcRn–mCherry in some of the HSA–AF488-positive tubular carriers can be explained by the additional presence of an endogenous FcRn pool in live BOECs, which was not visualised by this approach. After 40 min chase, tubular carriers continued to emerge from maturing macropinosomes, including FcRn–mCherry-single-positive tubules, indicating that the receptor can be sorted into tubular transport carriers independently from its ligand, HSA.

### Intracellular trafficking of HSA^H464Q^–AF488 in FcRn–mCherry live BOECs

**Fig. 9.**
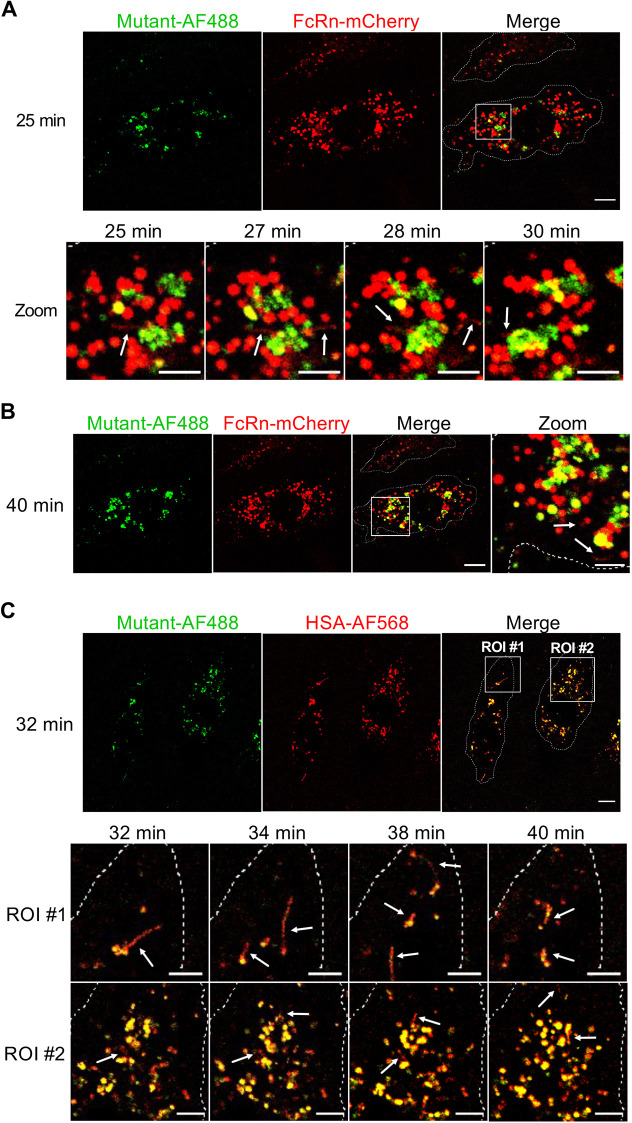
**HSA^H464Q^**–**AF488 is excluded from tubular carriers in FcRn–mCherry-transduced live BOECs.** (A,B) BOECs transduced with FcRn–mCherry (red) were pulsed with HSA^H464Q^–AF488 (Mutant–AF488, green) for 15 min at 37°C and the fluorescence signal chased for 40 min in live cells. The same field is shown throughout the chase in the live cells. Confocal images of live cells after 25, 27, 28 and 30 min chase (A) and after 40 min chase (B) are shown. White arrows indicate FcRn–mCherry-positive tubular transport carriers. (C) BOECs were pulsed with both HSA^H464Q^–AF488 (Mutant–AF488, green) and wildtype HSA–AF568 (red) for 15 min at 37°C, and the fluorescence signals chased for 40 min in live cells. Two ROIs were defined to follow the itinerary of single tubular transport carriers during the 32–40 min chase, as indicated. White arrows indicate wildtype HSA–AF568-positive tubular transport carriers. Cell boundaries are indicated by dotted lines. Images are representative of more than three independent experiments. Scale bars: 10 µm (merge); 5 µm (zoom).

To determine whether the sorting of endocytosed HSA molecules into tubular carriers is dependent on the interaction with FcRn, pulse-chase experiments were performed using the non-FcRn binding albumin variant HSA^H464Q^–AF488 (Fig. 9). Similar to HSA–AF488, after fluid-phase uptake, HSA^H464Q^–AF488 was localised to macropinosome structures, and with increasing chase time, it was observed to localise with FcRn–mCherry (Fig. 9A,B), demonstrating that the HSA mutant was localised to the same compartments as wildtype HSA during the early phase of the chase. After 25 min chase, tubular transport carriers positive for FcRn–mCherry (indicated by white arrows) were detected emerging from double-positive endosomal structures (Fig. 9A). Inspection of several frames within a 5 min timespan (25–30 min) revealed that HSA^H464Q^–AF488 was excluded from FcRn–mCherry-positive tubules, indicating sorting of FcRn–mCherry but not of HSA^H464Q^ molecules into these tubular transport carriers (Fig. 9A). After 40 min chase, the co-localisation of HSA^H464Q^–AF488 and FcRn–mCherry persisted in maturing macropinosomes, and FcRn–mCherry-positive tubular transport carriers (indicated by white arrows) continued to emerge from the double-positive macropinosomes, whereas HSA^H464Q^–AF488 remained excluded from these tubular carriers (Fig. 9A,B). These data suggest that FcRn–mCherry is actively partitioned into tubular carriers originating from maturing macropinosomes, and that the non-FcRn-binding albumin variant, HSA^H464Q^–AF488, is excluded from tubular carriers.

To further explore FcRn-dependent partitioning of HSA molecules into tubular transport carriers, a co-uptake experiment was performed using AF488-labelled HSA^H464Q^ and Alexa Fluor 568 (AF568)-labelled wildtype HSA and the fluorescence signals chased for up to 40 min (Fig. 9C). Both HSA variants co-localised to globular macropinosomes after endocytosis in the cell periphery for the first 25 min of the chase (Fig. S6). After 25 min chase, tubular transport carriers positive for HSA–AF568 (indicated by white arrows) were detected emerging from double-positive macropinosomes (Fig. 9C). With increasing chase time, these structures were observed more frequently, whereas HSA^H464Q^–AF488 remained excluded from tubular transport carriers.

Close inspection of ROIs after 32 min chase time revealed that these tubular carriers pinched off from the globular structures; however, fluorescence signals specific for HSA–AF568 were detected in tubular transport carriers, whereas HSA^H464Q^–AF488 was not partitioned into tubular structures (Fig. 9C). Analysis of 36 tubules emerging between 32 and 40 min chase showed that all tubules were positive for the WT HSA–AF568 signal, whereas only 3/36 (8%) were also positive for HSA^H464Q^–AF488. Comparison of single frames of the same ROIs at different chase times (between 32 and 40 min chase) indicated that the HSA–AF568-positive tubular transport carriers preferentially emerged in the cell periphery rather than in the perinuclear region of the cell and were directed towards the PM. These data collectively confirm the active sorting of wildtype HSA into tubular carriers from maturing macropinosomes and thereby show that HSA, but not the non-binding HSA mutant, is diverted from being transported to late endosomes and/or lysosomes.

## DISCUSSION

The vascular endothelium, consisting of a cellular monolayer lining the inner walls of blood vessels, is considered to be an important regulator of FcRn-mediated albumin homeostasis *in vivo* (Pyzik et al., 2019). Here, we established primary vascular endothelial cell lines isolated from peripheral blood (BOECs) to study FcRn–albumin cell biology in human primary endothelial cells. Our findings demonstrate that (1) BOECs express endogenous FcRn located predominantly to EEA1-positive early endosomal and macropinosome structures, but not to late endosomes, lysosomes or recycling endosomes; (2) BOECs are able to endocytose HSA efficiently via macropinocytosis; (3) the majority of endocytosed HSA by BOECs is not transported to the late endosomes and/or lysosomes; (4) the FcRn receptor is located within a highly dynamic, endosomal or tubular network, as revealed by transduction of FcRn–mCherry into BOECs; and (5) endocytosed HSA molecules are sorted into FcRn-positive tubular transport carriers, whereas the non-binding HSA variant HSA^H464Q^ is excluded from tubular structures. These findings demonstrate an FcRn-dependent rescue of endocytosed HSA in BOECs from transport to lysosomes.

Albumin endocytosis and FcRn-mediated recycling of albumin has been described in immortalised endothelial cell lines that overexpress exogenous FcRn (Grevys et al., 2018; Schmidt et al., 2017). Endothelial cells that have been used in previous studies are specialised endothelial cells that originated from the placenta, umbilical vein or dermal microvasculature (Lambot et al., 2006; Schmidt et al., 2017), and are phenotypically and functionally distinct from the endothelial cells of classic vasculature *in vivo* (Bouïs et al., 2001). A number of studies have exploited blood-borne progenitor cells to establish stable primary cell lines as an *in vitro* model of differentiated vascular endothelial cells (Asahara et al., 1997; Shi et al., 1998). Although several BOEC protocols are available, most of them either require extensive passaging before obtaining stable cell lines and high cell numbers or large volumes of peripheral blood (Lin et al., 2002; Martin-Ramirez et al., 2012; Ormiston et al., 2015). Here, we established a protocol for the generation of BOEC lines that only requires 30 ml of peripheral blood and allows the establishment of BOEC lines after a single passaging step. BOEC lines established by this protocol maintain proliferative capacities and show stable expression of the endothelial markers CD144 and vWF up to passage 14, exceeding the maximum passage number described by other BOEC protocols (Ecklu-Mensah et al., 2018; Lin et al., 2000). The primary BOEC lines are a suitable model for studying FcRn–albumin cell biology; BOECs express FcRn and the receptor is predominantly associated with enlarged EEA1-positive structures, presumably early endosomes and macropinosomes, and is absent from late endosomes. These findings are consistent with the intracellular localisation of transgenic FcRn in BMDMs (Toh et al., 2019) or immortalised cell lines (Chia et al., 2018; Ober et al., 2004; Ward et al., 2005). However, in immortalised endothelial cell lines, FcRn has also been reported to be located in Rab11-positive recycling endosomes (Ward et al., 2005), whereas FcRn was only weakly associated with recycling endosomes in BOECs, which reveals a phenotypic difference between primary BOECs, with high macropinocytic activity, from immortalised endothelial cells.

Our study showed that BOEC lines are capable of efficient albumin endocytosis by constitutive macropinocytosis. In contrast, albumin internalisation by immortalised cell lines at physiological pH is relatively inefficient as 1–4 h are required to obtain high levels of intracellular albumin (Chia et al., 2018; Grevys et al., 2018; Nilsen et al., 2020; Schmidt et al., 2017). Additionally, by use of the selective inhibitor EIPA, we showed that macropinocytosis is the major endocytic pathway for albumin uptake by BOECs. Albumin internalisation in rat pulmonary microvascular endothelial cells is proposed to be mediated by a surface receptor (gp60) and caveolin-dependent endocytosis (John et al., 2003; Li et al., 2013; Vogel et al., 2001), highlighting potential differences in albumin uptake and trafficking between cells from different species and/or endothelial origin. Macropinocytosis has been identified as the major albumin uptake pathway by primary macrophages (Commisso, 2019; Toh et al., 2019). Hence, as BOECs internalise HSA efficiently by fluid-phase macropinocytosis, BOECs provide an attractive system to investigate the trafficking pathways of FcRn–albumin in vascular endothelia.

To track the itinerary of endocytosed HSA, we performed pulse-chase studies in the presence of the endogenous FcRn alone and also with heterologously expressed mCherry-tagged FcRn to visualise the receptor. Importantly, the expression levels of FcRn–mCherry in BOECs were modest, indeed lower than endogenous FcRn protein levels, thereby minimizing the impact of receptor overexpression. Moreover, FcRn–mCherry showed a similar intracellular distribution as that of endogenous FcRn in BOECs, indicating that the intracellular location was compatible with that of the untagged receptor. Moreover, our previous studies showed that the FcRn–mCherry fusion protein was functionally active in BMDMs (Pannek et al., 2022; Toh et al., 2019), indicating that the fluorescently tagged FcRn is correctly located and functional.

Newly formed HSA-positive macropinosomes were initially not associated with FcRn; rather, between 10 and 20 min chase, macropinosomes were found to contain both HSA and FcRn, a time period also associated with acidification of early macropinosomes. The timing is relevant as an acidic pH is required for FcRn binding to albumin (Andersen et al., 2012). FcRn internalisation from the PM is presumably mediated via AP2 motifs on its cytoplasmic tail and clathrin-mediated endocytosis (Mahmoud et al., 2017).

**Fig. 10.**
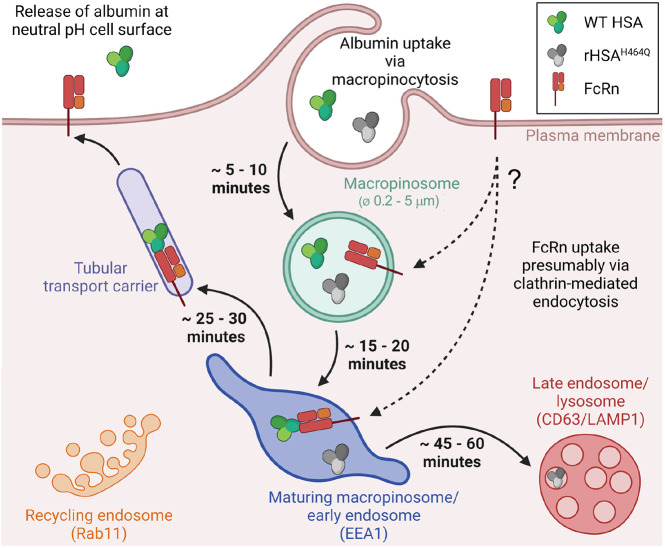
**Model of albumin trafficking in BOECs.** Cartoon showing the intracellular trafficking pathways of wildtype (WT) HSA (green) and the non-binding mutant HSA^H464Q^ (grey) mediated by FcRn (red/orange). Early macropinosomes are light green and acidic maturing macropinosomes are blue. The pathway of FcRn delivery to macropinosomes is not defined and could include delivery from the cell surface (as shown) or delivery from early endosomes and/or fusion of macropinosomes with early endosomes. The illustration was generated with BioRender.com.

Based on these findings, we propose the model shown in Fig. 10. HSA is internalised into BOECs by macropinosomes of 2–5 µm in diameter, which are initially devoid of FcRn. Over the subsequent 20 min period, the maturing early macropinosomes are acidified and become FcRn positive, either via fusion with FcRn-positive endosomes or FcRn internalisation from the PM by clathrin-mediated endocytosis and delivery to the maturing macropinosomes. The acidification of early macropinosomes in *Dictyostelium* is mediated by WASP regulation of V-ATPase (Buckley et al., 2016) and it is possible that a similar process might occur in mammalian endothelial cells. The emergence of HSA–AF488- and FcRn–mCherry-double-positive tubules was observed after 20–30 min uptake in live BOECs, a period during which acidification had occurred, which would allow for interaction of HSA and FcRn, as we previously demonstrated in BMDMs using biophysical techniques (Pannek et al., 2022). Newly formed macropinosomes then undergo extensive tubulation, which pinches off to form transport carriers that are transported to the periphery of the cell. Fluorescent albumin subsequently disappears from the cell, presumably as a consequence of cell surface delivery and exocytosis. In other cell types, newly formed macropinosomes have also been shown to tubulate extensively (Kerr et al., 2006) and, furthermore, such membrane tubules have been shown to transport cargoes to other intracellular destinations, including recycling to the PM (Bryant et al., 2007; Toh et al., 2019). Unfortunately, attempts to directly demonstrate HSA exocytosis by BOECs into the cell medium by either western blotting or enzyme-linked immunosorbent assays were inconsistent owing to a high background and lack of sensitivity. However, the velocities (∼0.4 µm/s) and morphology (5–7 µm long) of FcRn–mCherry- and HSA–AF488-positive tubular carriers resemble the phenotype of tubular transport carriers mediating FcRn-dependent recycling of endocytosed HSA in primary BMDMs (Toh et al., 2019), suggesting a similar function of the tubules observed in cultured BOECs. Candidates that might be involved in the formation of these tubular carriers include sorting nexins, namely SNX5 (Kerr et al., 2006), SNX17 (McNally et al., 2017; van Kerkhof et al., 2005) and SNX27 (Steinberg et al., 2013), Rab4, and retromer (Cullen and Steinberg, 2018) and retriever complexes (McNally et al., 2017). It will also be of interest to compare the macropinosome recycling pathway with the SNX BAR-mediated endosomal sorting tubules from early endosomes (van Weering et al., 2012). A key feature for comparison will be the precise diameter of the tubules, assessed by electron microscopy, as the diameter of the tubules is defined by the machinery responsible for driving the tubulation. Whereas the non-FcRn-binding HSA^H464Q^ variant is also co-localised in globular endosomal structures, only wildtype HSA is sorted into tubular transport carriers, a process presumably mediated by interaction with FcRn in live BOECs. In the absence of an interaction with FcRn, HSA^H464Q^ is then transported down the late endosomal/lysosomal pathway for degradation (Fig. 10).

In summary, our findings show the potential of BOECs to study macropinocytosis-mediated events and, using BOECs, we demonstrated that albumin is rapidly diverted from the endosome/lysosome pathway via a FcRn-mediated process into transport carriers to the cell surface. The findings also highlight the spatial, temporal and physical properties of the recycling system and they might be relevant issues for engineering ligands to exploit the FcRn-recycling system for extension of half-life *in vivo*.

## MATERIALS AND METHODS

### Reagents

Plasma-derived HSA (Sigma-Aldrich Merck) was labelled with Alexa Fluor 488 (AF488) NHS ester (succinimidyl ester) (Thermo Fisher Scientific, A-20000) or Alexa Fluor 594 (AF594) NHS ester (succinimidyl ester) (Thermo Fisher Scientific, A-37572), according to the manufacturer's protocol. Lysine-fixable Texas Red-conjugated 70 kDa dextran was purchased from Invitrogen (Thermo Fisher Scientific). Recombinant non-FcRn-binding albumin variant HSA^H464Q^ (Andersen et al., 2012) was generated and labelled with AF488 NHS ester (succinimidyl ester) (HSA^H464Q^–AF488). DQ ovalbumin (DQ-OVA) was purchased from Thermo Fisher Scientific and 4′,6 diamidino-2-pheynylindole (DAPI; D5942-5MG) was from Sigma-Aldrich, Merck.

### Antibodies and conjugates

The primary antibodies used were as follows: rabbit polyclonal antibodies to FcRn [FCGRT; HPA015130, Sigma-Aldrich Merck; 1:500 for immunofluorescence (IF) and western blotting (WB)]; mouse monoclonal antibodies against CD63 (sc5275, clone MX-49.129.5, Santa Cruz Biotechnology; 1:400 for IF); mouse monoclonal antibodies to CD144 [14-1449, clone 16B1, Thermo Fisher Scientific; 1:500 for IF, 1:400 for fluorescence-activated cell sorting (FACS)]; mouse monoclonal antibodies to CD31 (390-14-0311-82, Thermo Fisher Scientific; 1:500 for IF); mouse antibodies to GAPDH (AM4300, Thermo Fisher Scientific; 1:1000 for WB); mouse monoclonal antibodies to golgin-97 (A21270, clone CDF4, Thermo Fisher Scientific; 1:300 for IF); mouse monoclonal antibodies to EEA1 (610456, clone 14/EEA1, BD Biosciences; 1:400 for IF); mouse monoclonal antibodies to GM130 (610823, clone 35/GM130, BD Biosciences; 1:800 for IF); rabbit polyclonal antibodies to LAMP1 (ab24170, Abcam; 1:500 for IF), mCherry (ab183628, Abcam; 1:500 for IF and WB) and vWF (ab6994, Abcam; 1:400 for IF and FACS); and rabbit polyclonal antibodies to Rab11a (20229-1-AP, Proteintech, United Bioresearch; 1:1000 for IF).

The secondary antibodies used for IF were goat anti-rabbit IgG-Alexa Fluor 568, goat anti-mouse IgG-Alexa Fluor 568, goat anti-rabbit IgG-Alexa Fluor 488, goat anti-mouse IgG-Alexa Fluor 488, goat anti-mouse IgG-Alexa Fluor 647 and goat anti-rabbit IgG-Alexa Fluor 647; and antibodies used for WB were horseradish peroxidase-conjugated goat anti-rabbit IgG and goat anti-mouse IgG (all from Thermo Fisher Scientific). All secondary antibodies were used at 1:500 for FACS and IF, and 1:1000 for WB.

### Mice

FcRn^−/−^ mice, which harbour a KO allele of the mouse FcRn α-chain (*Fcgrt^tm1Dcr^*), and FcRn^−/−^ hFcRn Tg mice (hFcRn^Tg/Tg^) (lines 32 and 276) (Chaudhury et al., 2003; Petkova et al., 2006) were purchased from Jackson laboratory (Bar Harbor, ME, USA) and maintained as described (Toh et al., 2019). All experiments carried out on animals were approved by the University of Melbourne Animal Care and Use Committee (ethics ID: 1914968).

### Generation of BOEC cultures and maintenance

#### Collagen type I coating of culture vessels

An appropriate volume of collagen type I coating buffer containing 50 µg/ml collagen type I (Sigma-Aldrich, Merck) dissolved in 0.02 M acetic acid was added to cover the growth area of culture vessels and then incubated for at least 1 h at 37°C and 5% CO_2_. The coating buffer was aspirated and the culture vessels rinsed three times with PBS prior to their use for BOEC generation and maintenance.

#### Generation of BOECs

BOECs were generated essentially as described by Martin-Ramirez et al. (2012) and Ormiston et al. (2015) as follows. Peripheral human blood (30 ml) from healthy donors were collected in heparin tubes by the Volunteer Blood Donor Registry service at the Walter and Elizabeth Hall Institute (The University of Melbourne). Within 2 h after collection, PBMCs were isolated from human blood by discontinuous density gradient centrifugation using Ficoll-Paque Plus density gradient medium (GEHE17-1440-02, Bio-Strategy, Australia). The PBMC layer was carefully transferred into a 50 ml tube using a Pasteur pipette, the tube filled with PBS and the suspension was centrifuged for 7 min at 540 ***g*** and room temperature (RT). The supernatant was removed and cells were washed twice with 5 and 10 ml of Endothelial Cell Growth Medium 2 (EGM2) (Lonza, Australia) containing 18% (v/v) fetal bovine serum (FBS) (SAFC, Sigma-Aldrich, Merck), 1× GlutaMax supplement (Gibco, Thermo Fisher Scientific) and 100 U/ml penicillin and streptomycin (P4333 Sigma-Aldrich Merck) (BOEC medium) (7 min, 540 ***g***, RT). The cell pellet was resuspended in 20 ml BOEC medium, the cell suspension was adjusted to 2–2.5×10^6^ cells/ml, and 1–1.25×10^7^ cells were seeded into collagen type I-coated T-25 flasks and cultured at 37°C and 5% CO_2_. The medium was replaced after 48 h with fresh CO_2_-equilibrated BOEC medium and was subsequently changed after 2–3 days. Endothelial colonies were monitored daily using an inverted phase-contrast microscope and outgrowth of endothelial colonies was usually observed between days 14 and 28 of culture. Once radially spreading colonies reached a diameter of ∼1.5–2 cm, cells were trypsinised using TrypLE express enzyme (Thermo Fisher Scientific) and passaged into fresh collagen type I-coated T25-flasks (representing passage 1 of BOECs). The medium was replaced every 2–3 days with fresh CO_2_-equilibrated BOEC medium and cells passaged when monolayers reached 70–80% confluency.

The experiments carried out using human peripheral blood samples from healthy donors were approved by the Human Research Ethics committee, The University of Melbourne (ethics ID: 1749491). Informed consent was obtained from all blood donors.

#### Freezing and thawing BOECs

Confluent monolayers of BOECs were trypsinised using TrypLE express enzyme, washed with CO_2_-equilibrated BOEC medium and centrifuged for 5 min at 300 ***g*** and 4°C. The cell pellet was resuspended in BOEC freezing medium (95% FBS and 5% DMSO) (D2650, Sigma-Aldrich), the cell concentration adjusted to 3–7×10^5^ cells/ml and 1 ml aliquots of BOEC suspension transferred to cryovials and stored in vapour-phase liquid nitrogen.

Frozen cells were thawed in a 37°C water bath, washed with CO_2_-equilibrated BOEC medium and centrifuged for 5 min at 300 ***g*** and 4°C. The cell pellet was resuspended in fresh CO_2_-equilibrated BOEC medium, and cells were seeded into collagen type I-coated culture vessels with a density of at least 1×10^4^ cells/cm^2^ and incubated at 37°C and 5% CO_2_.

#### Maintenance of BOECs

Early passage (passage 1 or 2) or freshly thawed BOECs were maintained in CO_2_-equilibrated BOEC medium in collagen type I-coated culture vessels at 37°C and 5% CO_2_. Established BOEC lines (passage 3 or higher) were maintained in EGM2 containing 2% (v/v) FBS, 1× GlutaMax and 100 U/ml penicillin/streptomycin (C-EGM2) and in uncoated culture vessels at 37°C and 5% CO_2_. The medium was replaced with fresh BOEC or C-EGM2 medium every 2–3 days until 70–80% confluency. Semi-confluent monolayers were trypsinised using TrypLE express enzyme and cells were passaged into fresh culture vessels sustaining a density of at least 1×10^4^ cells/cm^2^. BOECs were regularly assessed for surface CD144 levels by FACS and only cell lines with CD144 levels >90% were used in experiments.

### Gene transfer protocols

#### Lipofectamine transfection of BOECs

Semi-confluent (60–80%) BOEC monolayers in 12-well plates or µ-dishes (ibidi, DKSH, Australia) were transiently transfected using Lipofectamine 3000 transfection reagent (Thermo Fisher Scientific) according to the manufacturer's protocol with slight adjustments. Briefly, 1.5 µl or 3 µl Lipofectamine 3000 was diluted in 50 µl Opti-MEM (Thermo Fisher Scientific) per well per dish and mixed well. In a fresh 1.5 ml tube, a DNA master mix containing 1–2 µg plasmid DNA per well was prepared by dilution in 50 µl Opti-MEM and addition of 2–4 µl P3000 reagent (Thermo Fisher Scientific). The DNA master mix was added to the diluted Lipofectamine 3000 and thoroughly vortexed, then incubated at RT for 10–15 min. 100 µl of the transfection mix per well/dish was added dropwise to the cells and plates incubated for 2–4 h at 37°C and 5% CO_2_, the Lipofectamine 3000-containing medium was then removed and replaced with fresh C-EGM2 medium and cell monolayers incubated for an additional 48 h at 37°C and 5% CO_2_.

#### Lentiviral transduction of BOECs

FcRn–mCherry lentivirus (LV) was generated as previously described (Pannek et al., 2022). Semi-confluent (60–80%) BOEC monolayers in 12-well plates or µ-dishes were transduced with FcRn–mCherry LV particles. Briefly, 10 µl concentrated LV was diluted in 1.2 ml EGM-2 medium containing 2% FBS and GlutaMax, the medium on monolayers replaced with the freshly prepared LV-containing medium, and the BOECs were incubated for 24 h at 37°C and 10% CO_2_. The LV-containing medium was then discarded, cells washed with PBS three times, fresh EGM-2 medium added, and the transduced cells incubated for an additional 24 h at 37°C and 5% CO_2_.

### Internalisation assays using BOECs

Confluent (80–100%) BOEC monolayers were washed three times with PBS and incubated for 30 or 60 min with 50 µg/ml fluorescently labelled transferrin (Tf–AF488 or Tf–AF568; Invitrogen Thermo Fisher Scientific), 100 µg/ml fluorescently labelled HSA (HSA–AF488 or HSA–AF568) or 500 µg/ml 70 kDa Dextran-Texas Red at 37°C and 5% CO_2_. Monolayers were then washed and cells were either fixed for confocal microscopy or prepared for flow cytometry as described below. For DQ-OVA uptake experiments, BOEC monolayers were incubated for up to 120 min in serum-free medium (SFM)-EGM2 containing 50 µg/ml DQ-OVA at 37°C and 5% CO_2_. At the indicated uptake times, live cells were prepared for flow cytometry or imaged by confocal microscopy at 37°C and 5% CO_2_ as described below.

### Pulse-chase assays

Confluent (80–100%) BOEC monolayers were washed three times with PBS and pulsed for 15 min (for live cells) or 30 min (for fixed cells) with 100 µg/ml of either HSA–AF488, HSA–AF568 or HSA^H464Q^–AF488 in SFM-EGM2 at 37°C and 5% CO_2_. Monolayers were washed three times with SFM-EGM2 to remove extracellular fluorophores, and intracellular fluorescence signals were chased for 60 min in SFM-EGM2 at 37°C and 5% CO_2_. At the indicated times, either cells were fixed or live cells were imaged by confocal microscopy at 37°C and 5% CO_2_ as described below. For DQ-OVA, BOEC monolayers were pulsed for 15 min with 50 µg/ml DQ-OVA in SFM-EGM2 at 37°C and 5% CO_2_ and washed three times with SFM-EGM2, and fluorescence signals were monitored over 120 min in SFM-EGM2 at 37°C and 5% CO_2_.

### PMA treatment

Confluent monolayers of BOECs were treated with C-EGM2 medium containing 80 nM PMA (P1585, Sigma-Aldrich, Merck) or 0.5% v/v DMSO (carrier control treatment) for 20 min at 37°C. Monolayers were washed and cells fixed with methanol as described below.

### EIPA treatment

A working solution of 10 mM EIPA (A3085, Sigma-Aldrich, Merck) was prepared in methanol. Confluent BOEC monolayers were treated in SFM-EGM2 containing the indicated concentrations of EIPA (0–200 µM), with methanol at 2% in all samples, for 60 min at 37°C and 5% CO_2_. The medium was removed, and subsequent internalisation assays were performed as described above in the presence of EIPA in SFM-EGM2. Cells were either prepared for flow cytometry or fixed for confocal microscopy.

### Cell fixation and permeabilisation

For PFA fixation, cell monolayers grown on coverslips were fixed in 4% (v/v) PFA for 10–15 min at RT and quenched in 50 mM NH_4_Cl/PBS for 10 min at RT. For trichloroacetic acid (TCA) fixation, cell monolayers grown on coverslips were fixed in 10% TCA for 15 min on ice and quenched in cold 30 mM glycine in PBS for 10 min at RT. For intracellular staining, fixed cells were also permeabilised with either 0.1% v/v Triton X-100 in PBS for 4 min at RT or 0.05% w/v saponin in PBS for 15 min at RT, and subsequently incubated in blocking solution (5% v/v FBS and 0.02% w/v sodium azide in PBS) for 30 min at RT to reduce non-specific binding prior to immunostaining.

For methanol fixation, cell monolayers were fixed in pre-cooled methanol for 5–10 min at −20°C and fixed cells were incubated in blocking solution (5% v/v FBS and 0.02% v/v sodium azide in PBS) for 30 min at RT before proceeding with immunostaining.

### Indirect immunostaining for confocal microscopy

Fixed and permeabilised cells on coverslips were incubated with primary antibodies diluted in blocking solution (5% v/v FBS and 0.02% v/v sodium azide in PBS) for 1 h at RT, washed three times with PBS and then incubated with secondary antibodies diluted in blocking solution for 30 min at RT. Cells were washed three times with PBS, cell nuclei stained with DAPI (Sigma-Aldrich) for 5 min, washed three times with PBS, rinsed once with milli-Q H_2_O and mounted in Mowiol [10% w/v Hopval 5-88 (475904 Calbiochem, Merck), 25% w/v glycerol, 0.1 M Tris].

### LysoTracker Red staining

Confluent monolayers of live BOECs were incubated with 1 mM LysoTracker Red DND-99 (Thermo Fisher Scientific) diluted 1:20,000 in C-EGM2 medium for 60 min at 37°C and 5% CO_2_. Cells were washed, fresh C-EGM2 medium was added and either live cell imaging or pulse-chase assays using HSA–AF488 were performed as described above.

### Flow cytometry

BOEC monolayers were trypsinised using TrypLE express enzyme, transferred to FACS tubes, washed with PBS containing 2% v/v FBS and 1 mM EDTA (FACS buffer), and pelleted at 300 ***g*** for 5 min at 4°C. The cell pellet was resuspended in 100 µl FACS buffer and incubated with primary antibodies for 30 min at 4°C, washed in FACS buffer and incubated with fluorophore-conjugated secondary antibodies for 30 min at 4°C in the dark. Cells were washed with FACS buffer, the cell pellet resuspended in DAPI staining buffer (1 µg/ml DAPI in PBS), incubated for 5 min at 4°C in the dark to stain dead cells, and washed with FACS buffer. Cells were analysed at a medium flow rate in an LSRFortessa flow cytometer (BD Biosciences), equipped with 405, 488, 561 and 640 nm lasers (BD Biosciences). Approximately 10,000 events were collected from cells resuspended in an appropriate amount of FACS buffer. Data were analysed using the FlowJo software (BD Biosciences).

### Confocal fluorescence microscopy of fixed cells

Images of fixed cell monolayers were acquired sequentially for multi-colour imaging on a laser confocal scanning microscope (Leica SP8 confocal imaging system, Leica Microsystems, Germany) using a 63× (1.4 NA) HC PL APO CS2 oil immersion objective and Leica HyD photodetectors. AF488 and GFP were excited using a 488 nm solid state laser; AF568, Texas Red and mCherry were excited with a 543 nm solid state laser; Alexa Fluor 647 (AF647) was excited with a 638 nm solid state laser; and DAPI was excited with a 405 nm DMOD laser. Leica HyD photodetectors were set to the collecting bandwidths as follows: 430–480 nm for DAPI, 500–500 nm for AF488 or GFP, 580–630 nm for AF568 or mCherry, and 650–700 nm for AF647. *Z*-stacks were acquired using a step size of 0.3 µm per *z*-slice. Single optical sections are shown, unless stated otherwise.

### Live-cell imaging

All live-cell imaging experiments were performed on an Olympus FV3000 laser scanning microscope using a 60× (1.2 NA) U Plan S Apo water immersion objective and cells were imaged at 37°C and 5% CO_2_. AF488, DQ-OVA and GFP were excited using a 488 nm solid-state laser diode. AF568, LysoTracker Red and mCherry were excited using 561 nm solid-state laser diode. Fluorescence signals were collected with GaAsP detectors using the following bandwidths: 500–540 nm for AF488, DQ-OVA and GFP; and 590–650 nm for AF568, LysoTracker Red and mCherry. *Z*-stacks were acquired using a step size of 0.5 µm per section. Single optical sections of live-cell images are shown, unless stated otherwise.

### Macropinosome quantitation assay

BOECs were imaged by confocal microscopy and *z*-stacks of whole cells were acquired. Macropinosome-like structures were counted and the fluorescence levels of endocytosed HSA–AF488 and Tf–AF568 per cell were calculated using an adapted ImageJ-based macropinosome quantitation assay established by Wang et al. (2014). Maximum projections of *z-*stacks were generated, and boundaries of single cells defined as ROIs using DAPI and the background signal of the Tf–AF568 fluorescence channel. The Renyi Entropy threshold algorithm (https://imagej.nih.gov/ij/plugins/multi-thresholder.html) was used to identify HSA-positive endosomal structures and the Iso Data threshold algorithm (https://imagej.nih.gov/ij/plugins/multi-thresholder.html) was used to identify transferrin-positive endosomal structures. To quantify the number of HSA-positive macropinosomes per cell, particles with an area of 0.2–20 µm^2^, corresponding to globular structures with a diameter of ∼0.5–5 µm, within single ROIs were counted. Fluorescence intensities of intracellular HSA–AF488 and Tf–AF568 per cell were estimated by quantification of the integrated fluorescence intensities of the fluorescence signal limited to the thresholded structures within each ROI.

### Quantification of intracellular HSA fluorescence levels

The maximum projections of acquired *z*-stacks of fixed cells were generated and boundaries of single cells defined as ROI using DAPI and background fluorescence of an antibody-specific channel. Intracellular HSA fluorescence levels were calculated using whole *z-*stacks by quantification of the HSA-specific integrated fluorescence densities within single ROIs. HSA-positive endosomal structures were identified using ImageJ, following which integrated densities were calculated for each optical section of the *z*-stacks within single ROIs and summed to obtain total intracellular HSA fluorescence levels per cell. The mean total intracellular HSA level at 0 min chase for each experiment was set to 1 and data from up to six independent experiments were pooled. Intracellular fluorescence quantification was performed using ImageJ software.

### Quantification of co-localisation

Pulse-chase assays with BOEC monolayers using fluorescently labelled HSA derivates were performed as described above and imaged by confocal microscopy. The maximum projections of acquired *z*-stacks of fixed cells were generated and boundaries of single cells defined as ROI using the DAPI and the background fluorescence of an antibody-specific channel. Co-localisation was calculated within single ROIs of each section of the *z*-stack using the Coloc 2 plugin for Image J. Thresholds were calculated by Costes' auto-threshold regression and significance of obtained co-localisation parameters evaluated by Costes' randomisations (*n*=100) (Costes et al., 2004). The Costes' *P*-value for all calculated co-localisation parameters was 1.00. Pearson's coefficients were calculated for two fluorescence channels to assess their co-localisation to endosomal structures (Manders et al., 1992).

### Immunoblotting

Cells were extracted and analysed by immunoblotting as previously described (Toh et al., 2019). Chemiluminescence was detected using a ChemiDoc system (Bio-Rad) and the blots were auto-grey scaled with the Image Lab software (Bio-Rad).

### Statistical analysis

Raw data were processed using Excel and statistically analysed using GraphPad Prism. Error bars are graphically represented as s.e.m. Statistical significance was determined using the Fisher's least significant difference (LSD) test. *P*-values below 0.05 were considered statistically significant and are indicated as follows: ns, not significant, *P*>0.05; *P≤0.05; **P≤0.01; ***P≤0.001; ****P≤0.0001.

## Supplementary Material

10.1242/joces.260912_sup1 Supplementary information
