# Supplementary material for: The endosomal system of primary human vascular endothelial cells and albumin–FcRn trafficking
Source: J Cell Sci. 2023 Aug 11;136(15):jcs260912. doi: 10.1242/jcs.260912 (PMC10445748; doi:10.1242/jcs.260912)
Supplement: Supplementary information [file joces-136-260912-s1.pdf]

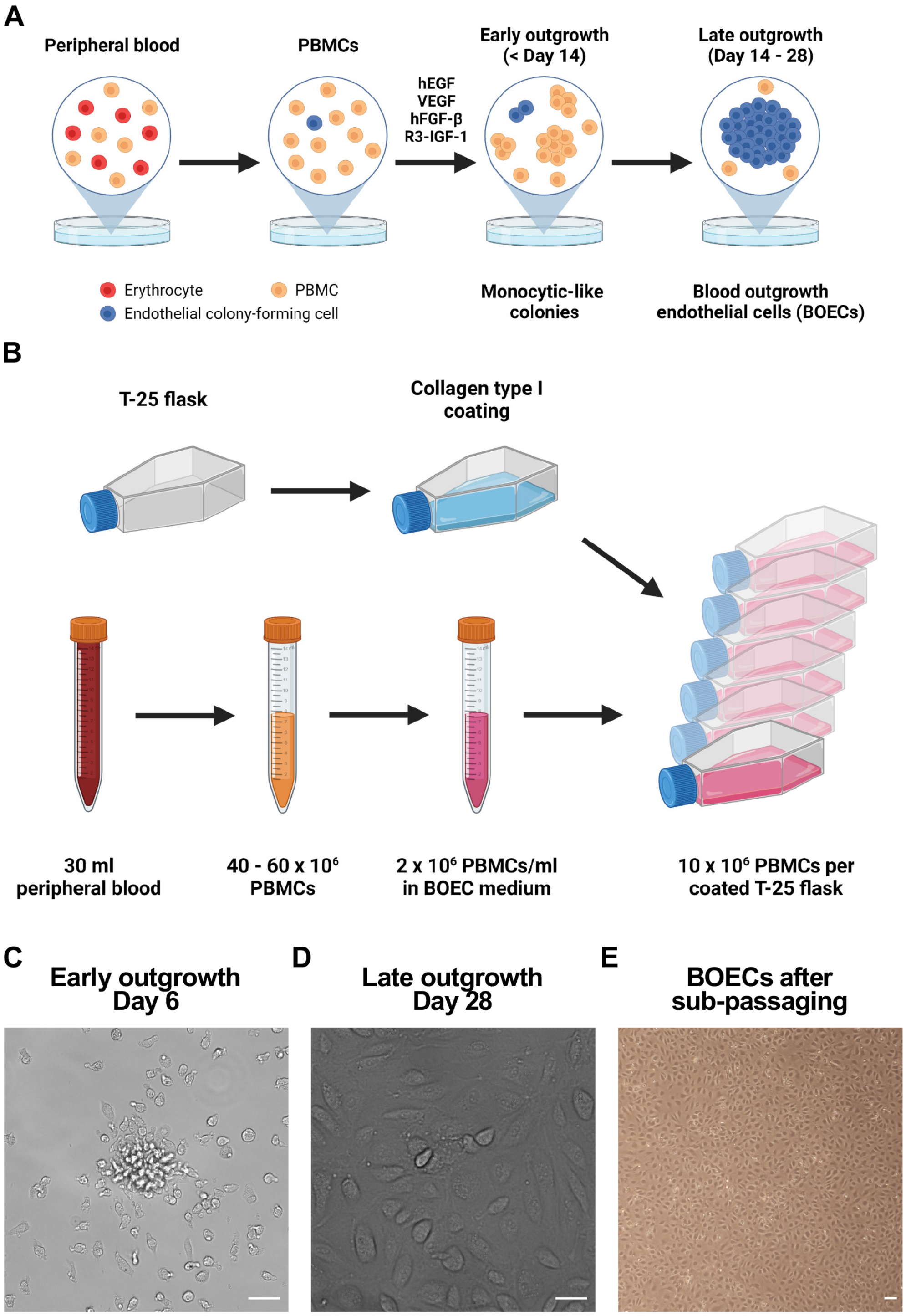

Fig. S1. Generation of BOEC and morphology of blood outgrowth endothelial colonies

(A) Principle of the generation of BOEC lines from human peripheral blood. **(B)** Protocol for the generation of stable BOEC lines from PBMCs using Collagen-coated T-25 flasks. The generation protocol was adapted from Martin-Ramirez *et al.* and Ormiston *et al.* (Martin-Ramirez *et al.*, 2012; Ormiston *et al.*, 2015). **(C-D)** Brightfield microscopy images of early **(C)** and late **(D)** outgrowth cell colonies on day 6 and 28 of culture, respectively. Scale bars represent 50 μm for C and 25 μm for D. **(E)** Brightfield microscopy image of a confluent established BOEC line exhibiting cobblestone-like morphology after sub-passaging. Scale bar represents 100 μm. Figures were created with BioRender.com.

Journal of Cell Science • Supplementary information

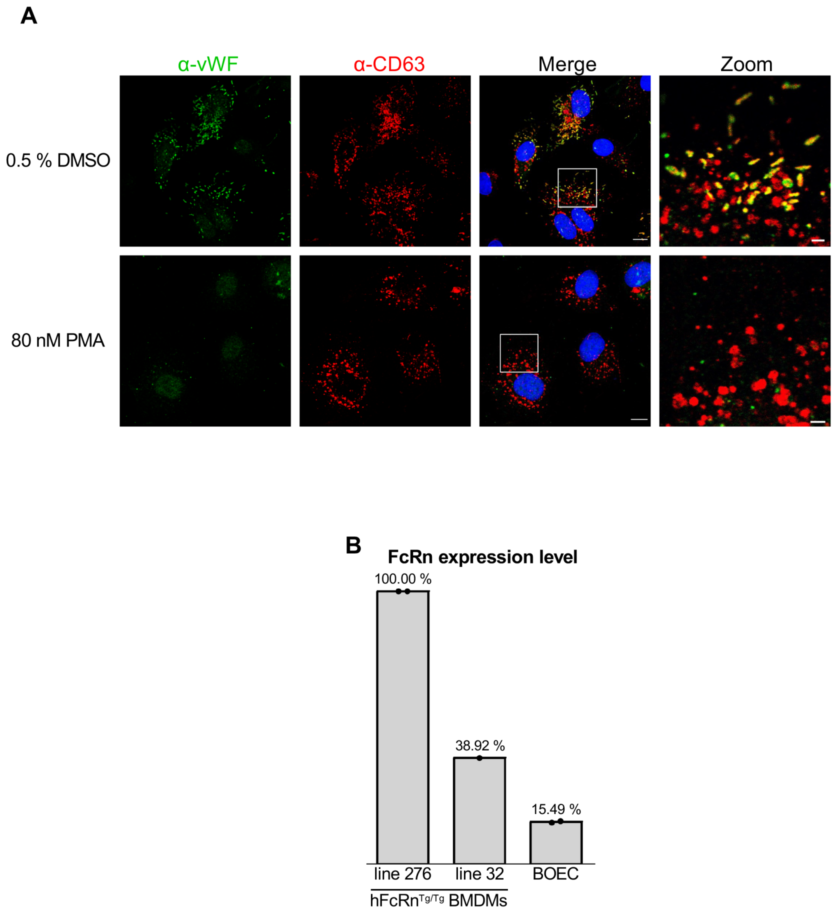

Fig. S2. Characterisation of BOEC cultures (extension of Figure 1)

A. *Release of vWF-positive Weibel-Palade bodies in BOECs upon PMA treatment (extension of Figure 1B).* Cultured BOECs were treated with 0.5 % DMSO (carrier control) or 80 nM phorbol- 12-myristate-13-acetate (PMA) for 20 minutes and subsequently fixed with MeOH. Cells were co-stained with anti-vWF (green) and CD63 (red) antibodies and nuclei were visualised using DAPI (blue). Scale bars represent 10 µm (Merge) or 2 µm (Zoom).

(B) *Densitometric analysis of western blot to detect human FcRn a-chain (Extension of Figure 1E)*. FcRn levels were normalised to detected GAPDH levels and the quantified FcRn level in lysates from hFcRn$^{Tg/Tg}$ line 276 BMDMs was set to 100 %. Percentages of FcRn levels are shown above the respective columns. FcRn levels for hFcRn$^{Tg/Tg}$ line 276 cells and BOECs were quantified from two independent experiments, whereas the FcRn level in hFcRn$^{Tg/Tg}$ line 32 cells was quantified in a single experiment.

Journal of Cell Science • Supplementary information

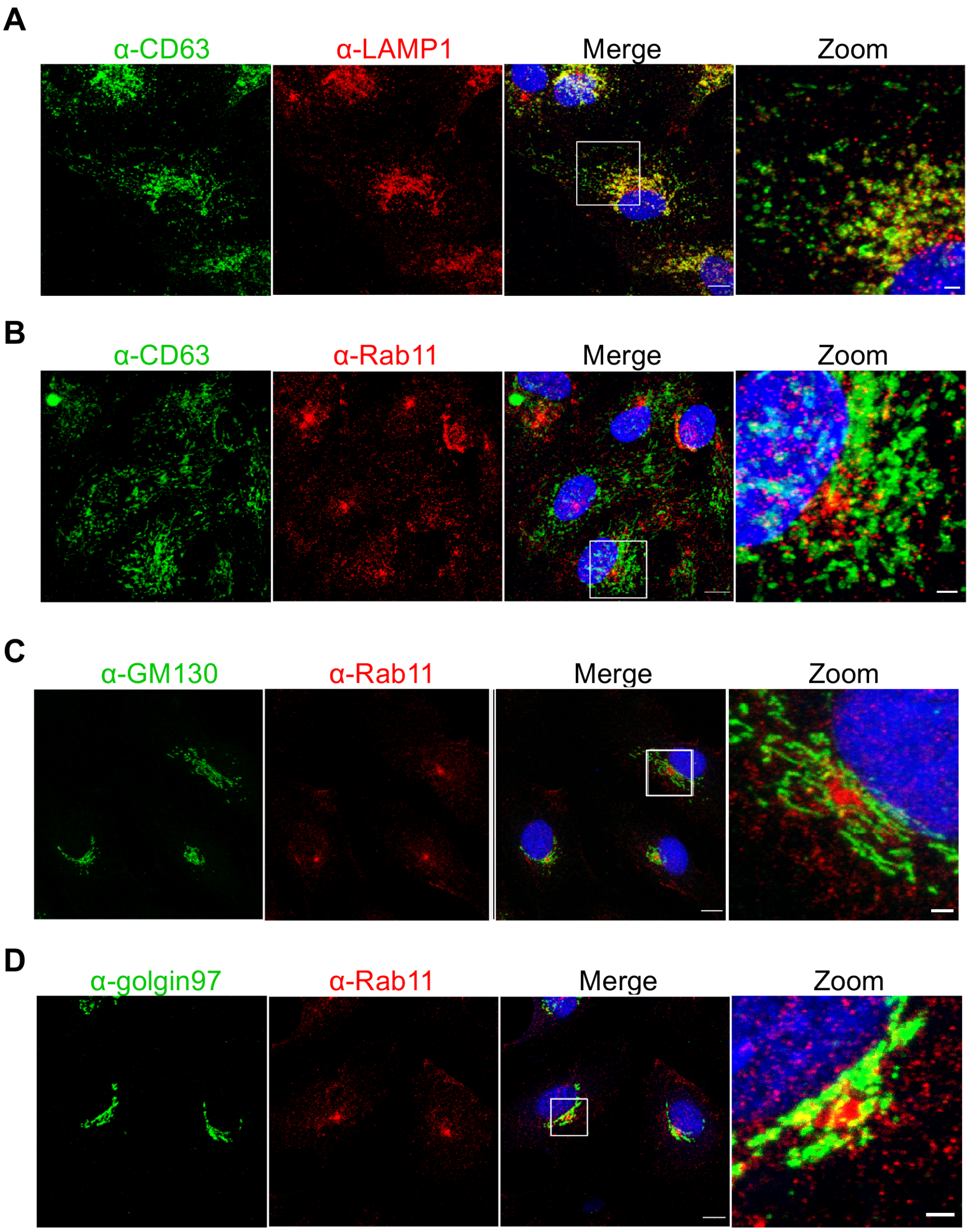

Fig. S3. Distribution of late endosomes and recycling endosomes in cultured BOECs (extension of Figure 1)

Cultured BOECs were fixed with MeOH and either co-stained for CD63 (green) and LAMP1 (red) **(A)** or CD63 (green) and Rab11a (red) **(B),** GM130 (green) and Rab11a (red) **(C)** or golgin97 (green) and Rab11a (red) (**D**), using specific antibodies. Nuclei were visualised using DAPI (blue). Scale bars represent 10 μm or 2 μm (Zoom).

Journal of Cell Science • Supplementary information

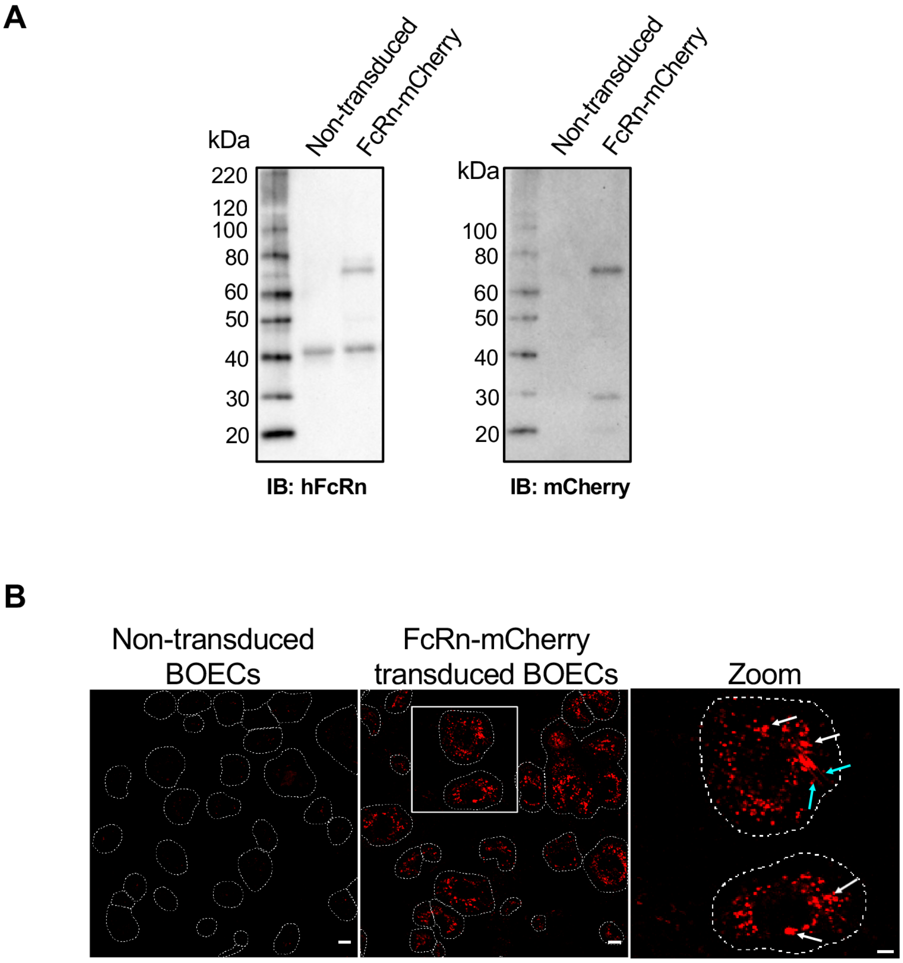

Fig. S4. Lentiviral transduction of FcRn-mCherry in cultured BOECs
BOECs were either untreated or transduced with 10 µl of lentivirus containing pFUGW-B2M-FcRn-mCherry (red) for 24 hours, monolayers washed and incubation for an additional 24 hours. **(A)** Transduced BOECs were lysed and proteins resolved by SDS-PAGE using a 4-12 % gradient gel. Separated proteins were transferred onto PVDF membranes and probed with an antibody against human FcRn and HRP-conjugated secondary antibody. Chemiluminescence was detected using a ChemiDoc™ system. After detection, membranes were stripped and reprobed with an anti-mCherry antibody and HRP-conjugated secondary antibody to detect mCherry-specific chemiluminescence. The marker line (M) was loaded with MagicMark™ Protein Standard. (**B**) Transduced live BOECs were imaged at 37 °C and 5 % CO_2 with a FV3000 Olympus confocal microscope. Enlarged FcRn-positive endosomal structures are indicated by white, tubular extensions by sky blue arrows. Cell nuclei were visualized using DAPI (blue). Scale bars represent 10 µm or 5 µm (Zoom).

Journal of Cell Science • Supplementary information

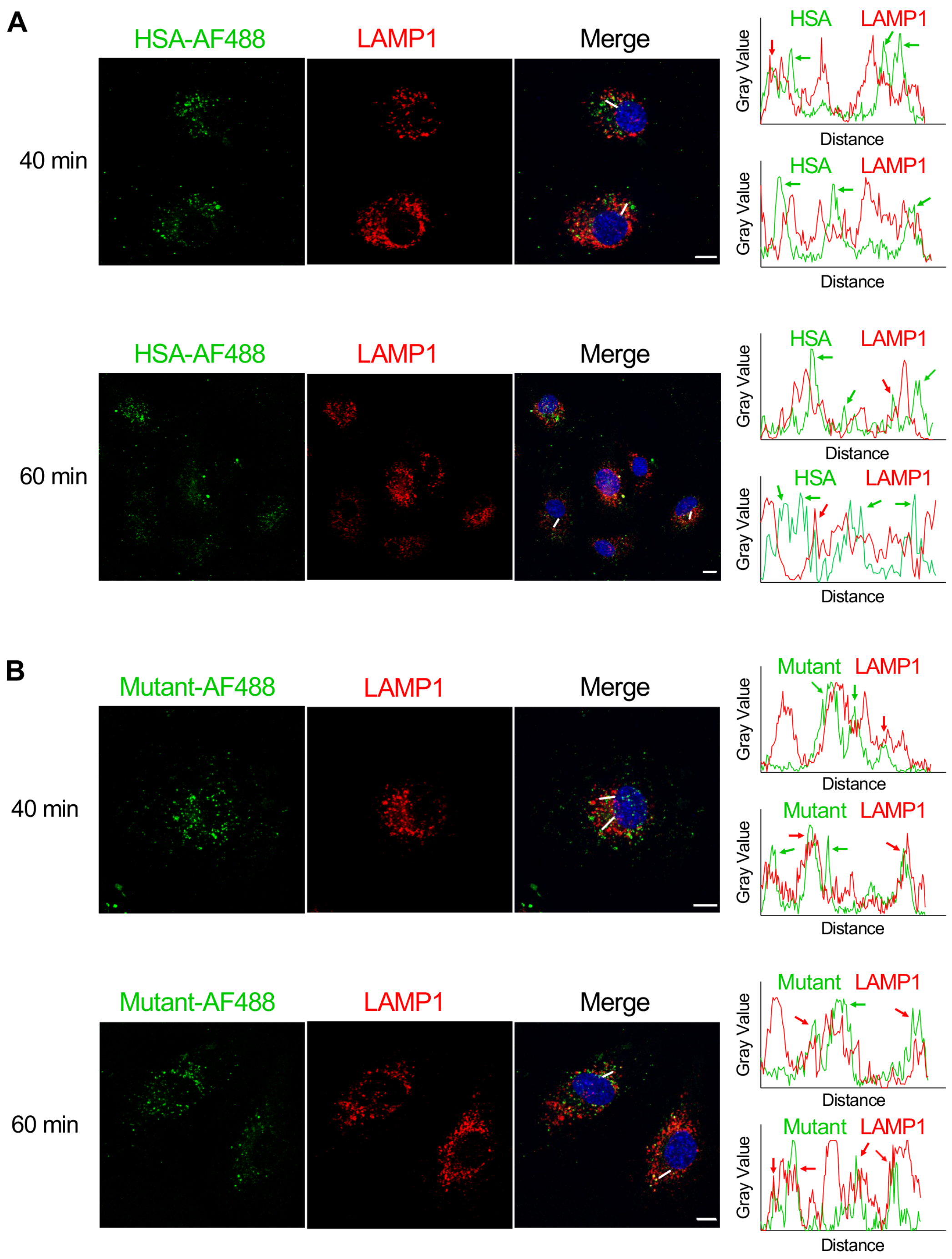

Fig. S5. Co-localisation of internalised HSA and LAMP1-positive endosomal structures in fixed BOECs (extension of Figure 6)

BOECs were pulsed with HSA-AF488 (green) **(A)** or non-FcRn binding HSA^{H464Q} (Mutant AF488, green) **(B)** for 30 minutes at 37 °C. Monolayers were washed and the fluorescent signal chased for 40 or 60 minutes at 37 °C. After the chase, cells were fixed with PFA and stained for LAMP1 (red). Nuclei were visualised using DAPI (blue). The profiles of two line scans per image are shown for both fluorophores. The localisation of the respective line scans is indicated by white lines. Images represent maximum projections of whole cell z-stacks. Scale bars represent 10 μm.

Journal of Cell Science • Supplementary information

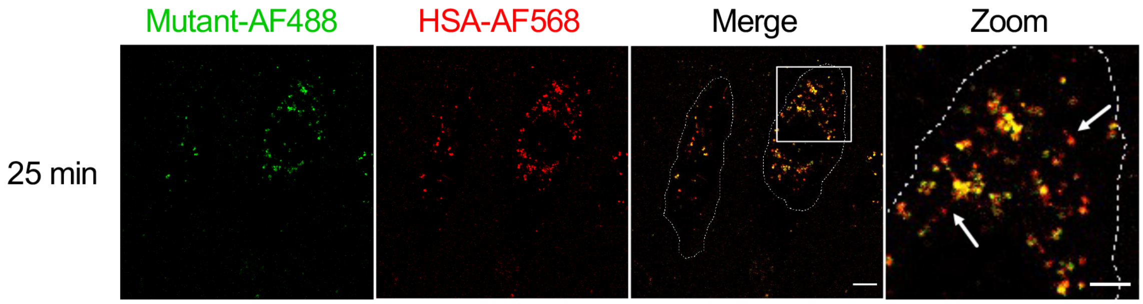

Fig. S6. Trafficking of HSA^{H464Q}-AF488 and HSA-AF568 in live BOECs (extension of Figure 9B)

BOECs were pulsed with both HSA^{H464Q}-AF488 (Mutant-AF488, green) and wildtype HSA-AF568 (red) for 15 minutes at 37 °C and the fluorescence signals chased for 40 minutes in live cells. Shown are the confocal microscopy images of live cells were taken after 25 minutes chase. See Figure 9 for subsequent chase times. Cell boundaries are indicated by dotted lines. Scale bars: 10 μm (merge), represent 5 μm (zoom).

Figure S7

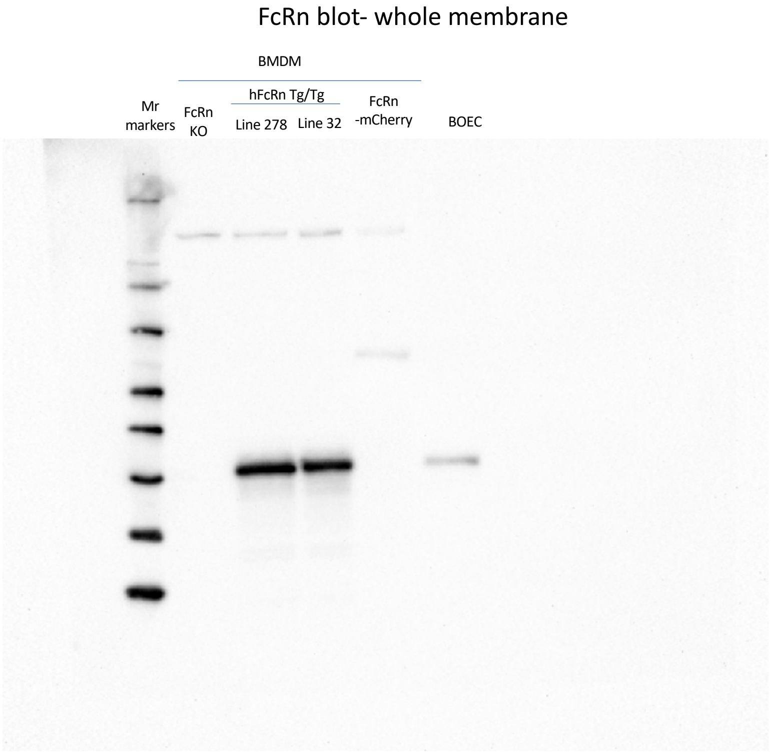

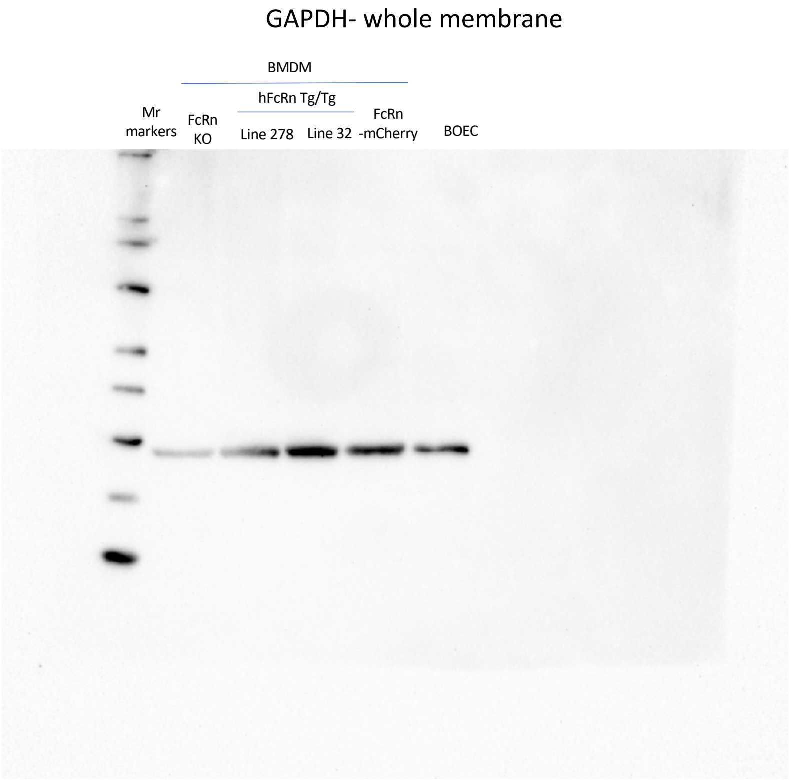

Fig. S7. Blot transparency – whole membrane blots (extension of Figure 1E)

Journal of Cell Science • Supplementary information

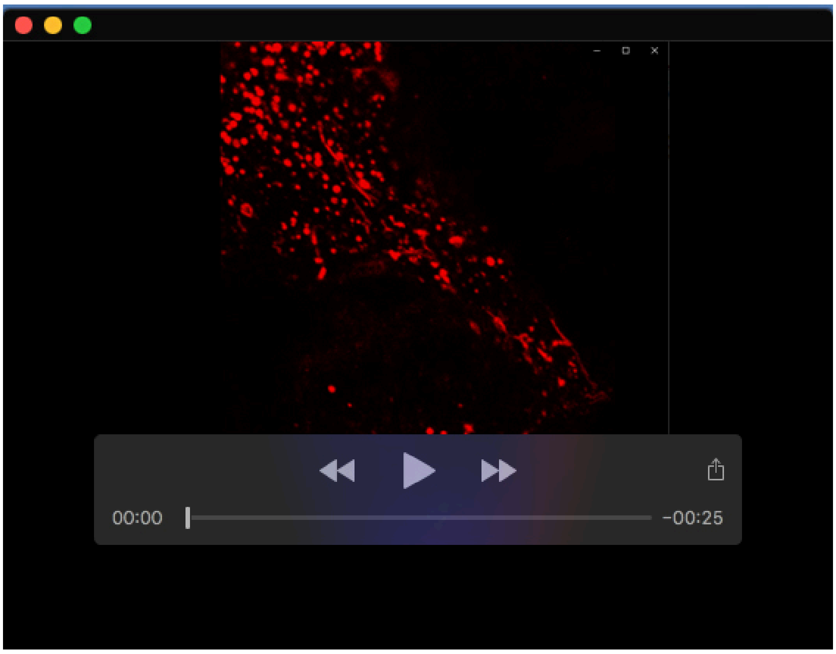

Movie 1. FcRn-mCherry dynamics

BOECs were transduced with recombinant B2M-FcRn-mCherry lentivirus for 24 hours, monolayers washed and cells incubated for an additional 24 hours. Live BOECs were imaged at 37 °C and 5 % CO_2 with a FV3000 Olympus confocal microscope. The delay between image capture was 5.84 sec.

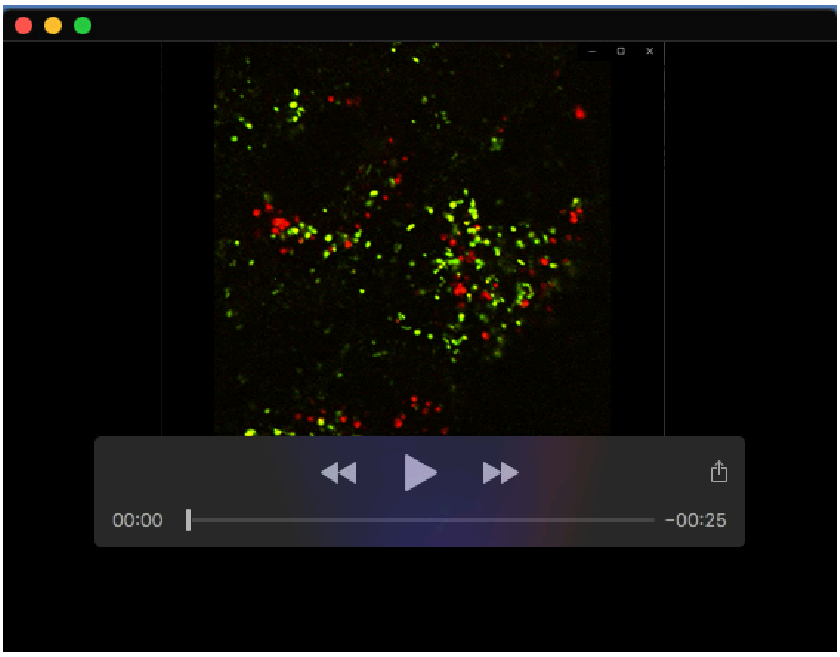

Movie 2. Localisation of endocytosed HSA-AF488 to LysoTracker Red-positive compartments in live BOECs

Cultured BOECs were stained with LysoTracker™ Red DND-99 (red) for 60 minutes at 37 °C and subsequently pulsed with HSA-AF488, green for 15 minutes at 37 °C and then chased. Live cells were imaged at 37 °C and 5 % CO_2 using an Olympus FV3000 confocal fluorescence microscope from 30 minutes chase with a 20 second delay between image capture.

Journal of Cell Science • Supplementary information

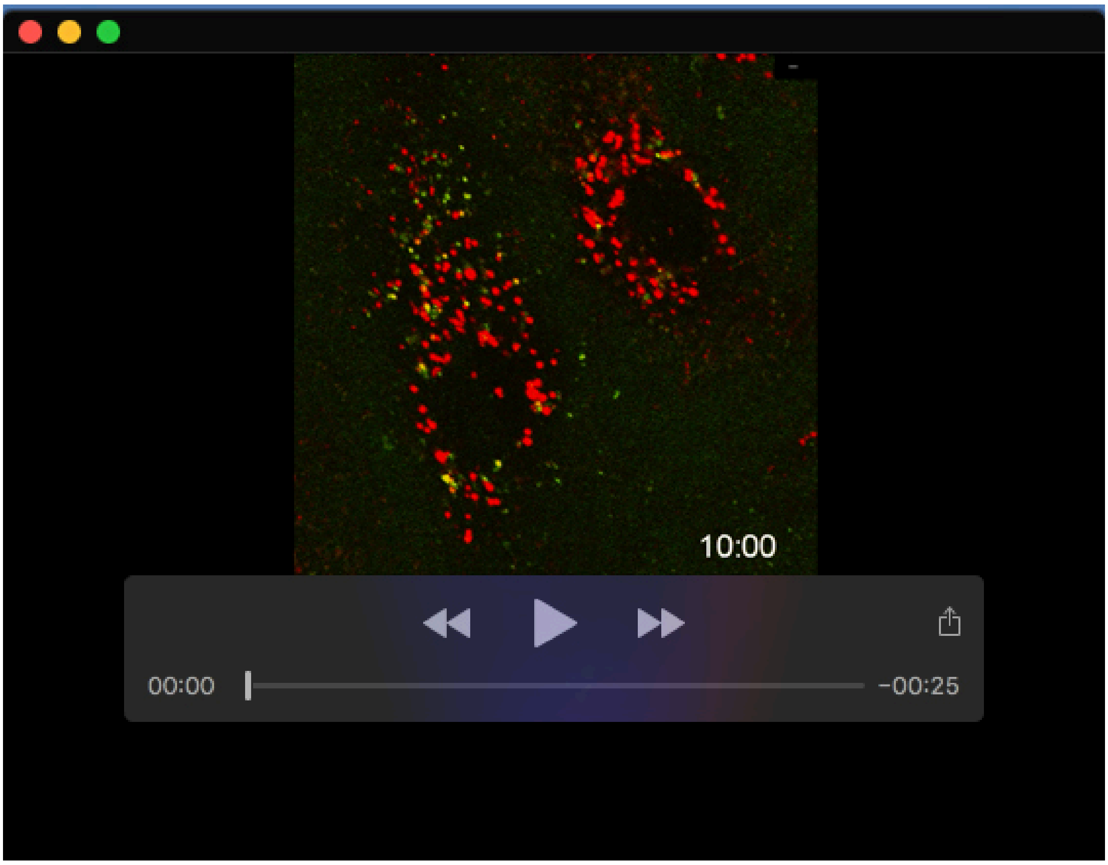

Movie 3. Intracellular trafficking of HSA-AF488 in FcRn-mCherry transduced live BOECs. BOECs transduced with FcRn-mCherry (red) were pulsed with HSA-AF488 (green) for 15 minutes at 37 °C and the fluorescent signal chased for 40 minutes in live cells and live BOECs were imaged at 37 °C and 5% CO_2 with a FV3000 Olympus confocal microscope. The video starts at 10 minutes of chase with a 20 second delay between image capture.

Journal of Cell Science • Supplementary information
