# Supplementary material for: The endosomal system of primary human vascular endothelial cells and albumin–FcRn trafficking
Source: J Cell Sci. 2023 Aug 11;136(15):jcs260912. doi: 10.1242/jcs.260912 (PMC10445748; doi:10.1242/jcs.260912)
Supplement: Supplementary file 1 [file joces-136-260912_review_history.pdf]

The endosomal system of primary human vascular endothelial cells and albumin-FcRn trafficking

Andreas Pannek, Janine Becker-Gotot, Steven K. Dower, Anne M. Verhagen and Paul A. Gleeson
DOI: 10.1242/jcs.260912

Editor: Jennifer Lippincott-Schwartz

Review timeline

Original submission:	22 December 2022
Editorial decision:	6 March 2023
First revision received:	12 June 2023
Accepted:	26 June 2023

Original submission

First decision letter

MS ID#: JOCES/2022/260912

MS TITLE: The endosomal system of primary human vascular endothelial cells and albumin-FcRn trafficking

AUTHORS: Andreas Pannek, Janine Becker-Gotot, Steven K. Dower, Anne M. Verhagen, and Paul A. Gleeson
ARTICLE TYPE: Research Article

We have now reached a decision on the above manuscript.

To see the reviewers' reports and a copy of this decision letter, please go to: https://submit-jcs.biologists.org and click on the 'Manuscripts with Decisions' queue in the Author Area. (Corresponding author only has access to reviews.)

As you will see, the reviewers raise a number of substantial criticisms that prevent me from accepting the paper at this stage. They suggest, however, that a revised version might prove acceptable, if you can address their concerns. If you think that you can deal satisfactorily with the criticisms on revision, I would be pleased to see a revised manuscript. We would then return it to the reviewers.

Please ensure that you clearly highlight all changes made in the revised manuscript. Please avoid using 'Tracked changes' in Word files as these are lost in PDF conversion.

I should be grateful if you would also provide a point-by-point response detailing how you have dealt with the points raised by the reviewers in the 'Response to Reviewers' box. Please attend to all of the reviewers' comments. If you do not agree with any of their criticisms or suggestions please explain clearly why this is so.

© 2023. Published by The Company of Biologists under the terms of the Creative Commons Attribution License (https://creativecommons.org/licenses/by/4.0/).

Reviewer 1

Advance summary and potential significance to field

This manuscript by Pannek et al addresses the physiologically prevalent and important pathways for the uptake, recycling and recovery of human serum albumin in endothelial cells. While this group and others have comprehensively outlined albumin recovery in macrophages, they now address albumin recycling in progenitor-derived ‘blood outgrowth endothelial cells’ as a tractable model of the endothelium in the general vasculature. Their results here bear some difference compared to HSA recycling in other published endothelial cell lines and in macrophages. The capacity of BOEC for constitutive macropinocytosis is highly relevant in this context and important to document.

The results here show the uptake of HSA by macropinocytosis and intersection with the recovery receptor, internalised FcRn, in macropinosomes/early endosomes and then return to the plasma membrane (PM) in ‘tubular transport carriers’, which are quite distinctively shown in live imaging here, but not identified by other markers or other cargo at this stage. The manuscript uses high level live and fixed imaging, and uses mutant ligand and virally transduced cells for these experiments. The approaches are straightforward and although not elaborate, they are entirely appropriate for tracking this pathway.

The data show convincingly and interestingly that FcRn does not transit the classic Rab11 recycling endosomes in BOEC. However the possible identity of the alternative tubular transport carriers for recycling to the PM (as in Figure 8) is not discussed. How do these structures or the HSA trajectories measured here compare to the ‘fast recycling pathway’ from macropinosomes described earlier by these authors and other groups?

The internalisation pathway for FcRn is not identified here but conjectured to be (or include) clathrin vesicles. It might be of interest in the future to track FcRn uptake with 10 kDa dextran and to measure intersection with 70 kDa dextran in macropinosomes. This arm of the pathway is less important for the current story than the recycling ‘exit’ pathway for FcRn and HSA.

Overall, this is a clear and interesting story supported by high quality data.
Albumin recovery pathways are physiologically vitally important, yet not sufficiently addressed in the cell biology literature. This manuscript is a valuable addition to the this field by documenting this endothelial version of the recovery pathway. Future studies will no doubt pick up further identification of the compartments and carriers involved.

Comments for the author

Minor comments to be addressed.
The BOEC cells are presented as a novel feature of the manuscript, and their generation is nicely described in Figure S1 and on page 5. However, it also would be helpful to have more information on the reproducibility of these cell phenotypes. Brief comments about the reproducibility of the cell growth appearance and endothelial markers in cells derived from different batches of blood/PBMCs , and over different passages, would be helpful. The text often refers to staining in ‘large globular ‘or ‘globular endosomal structures ‘ (eg with reference to Figures 6 and 7) in describing the images. It might be less cumbersome and more informative for the reader to simply assign them the labels of macropinosomes or of maturing macropinosomes according to the model in Figure 8. The continuity of the mature macropinosome/early endosome is sufficiently well defined and accepted in the literature. The reference cited on pg 11 as Wang et al 2014 does not appear in the list of references.

Label axis on Fig 4D as HSA fluorescence since rest of figure is DQ-OVA
The discussion should mention or speculate about possible markers for the tubular transport carriers and compare/contrast this with fast recycling pathways in the literature.

© 2023. Published by The Company of Biologists under the terms of the Creative Commons Attribution License (https://creativecommons.org/licenses/by/4.0/).

Reviewer 2

Advance summary and potential significance to field

We have read and discussed "The endosomal system of primary human vascular endothelial cell and albumin-FcRn trafficking," by Pannek et al. This work aims to characterize the endocytic transport and recycling of human serum albumin by FcRn in primary-derived blood outgrowth endothelial cells. The main conclusions of the paper are that HSA is internalized by fluid-phase macropinocytosis following which FcRn facilitates its recycling by packaging into tubules that presumably are transported to the plasma membrane where the neutral pH of the extracellular space releases the HSA from the FcRn. Overall, this is an important study that contributes to the understanding of serum albumin homeostasis.
However, in its current form, this study is challenging to read, with many of the key findings relegated to supplemental information. Additionally, the arrangement and display of the data could be substantially improved to focus on the illustrating the key points.

Comments for the author

Major concerns:
It is difficult to sort out what are bonified macropinosomes versus some other type of early endosomal compartment. The morphology of the endosomes in figures 1F and 1H appears more consistent with sorting endosomes and docked early endosomes rather than the smooth macropinosomes we are accustomed to seeing in macrophages and cancer cells. Part of this is likely due to fixation and staining strategies. Figure 3D is critical. This would likely be much clearer as a live-cell experiment. In which the dextran and HSA could be visualized early e.g. 5-10 min pulse with dextran + HSA, wash and image for another 10-20 min. Fixation of macropinosome contents is difficult and leads to the punctate morphology on the periphery of the macropinosome. The data in Fig. 3 are consistent with this. In many cell types like macrophages the 30 min. interval used would be very late – the vast majority of macropinosomes would have already undergone fusion with other early endosomes and had many components trafficked away.

The manuscript is unclear as to what compartment FcRn predominantly resides in. It is sometimes called a macropinosome, however, there is no data provided illustrating that a fluid phase marker resides within them. Moreover, they invariably look much larger than the HSA and dextran containing compartment. An experiment clarifying this point would be very helpful. It seems more consistent that FcRn is residing in a particular compartment.

The tubules appear to be independent of macropinocytosis – as FcRn alone seems to do this constitutively. Does EIPA inhibit tubule formation? Does EIPA disburse FcRn to the cell surface?
The diagram in Figure 8 needs some careful consideration. From the data provided there is little evidence that FcRn is being brought in on macropinosomes coincident with HSA. Rather it appears that FcRn endosomes interacts increasingly with with HSA endosomes at later times 40 min chase (Fig. 6) suggesting it is trafficked there. Figure 7A seems to indicate that only one MP out of many FcRn compartments dots co-localizes with HSA488 early, but this increases over time.

The description of the results feels like it relies equally on supplemental figures and regular figures to string the story together. This really makes the reader do a lot of work. I would suggest a careful consideration of what data is critical for the story and only relegate truly supplemental information to the supplement. For example, the experiments in Fig. S5 indicating that FcRn is not in the recycling endosome are critical, especially given that the morphology of the compartment does not appear to be that of a typical macropinosome. Yet figure 2 uses an entire page to illustrate the dynamics of a tubule – which are frequently associated with both macropinosomes and sorting endosomes. A little more care in the figure flow would make the manuscript more readable. Please consider making color overlay using magenta and green to make the work more accessible to color-blind readers.

https://www.ascb.org/science-news/how-to-make-scientific-figures-accessible-to-readers-with-color-blindness/

What about a transwell experiment to show the pumping of HSA from basal to apical surface?

© 2023. Published by The Company of Biologists under the terms of the Creative Commons Attribution License (https://creativecommons.org/licenses/by/4.0/).

Minor comments:

This sentence on page 5 is unclear:
"In this study, we adapted the protocol for the generation of BOECs to meet the requirements for investigating FcRn-albumin cell biology in vascular endothelial cells (Fig. S1B), in particular a protocol which allowed a reliable and fast establishment of endothelial cell lines with high proliferative properties."

The MIP representation in Figure 2A does't seem to add much and the tubules are difficult to see. I would suggest dropping it.

Figure 4 appears to contradict the interpretation in the results (that recycling is the predominant mode) The DQ-OVA data shows heavy depredation beginning at 40 min.
"In particular, the reduction in HSA fluorescence per cell during the first 40 minutes of chase, suggests that recycling may be responsible for the loss of fluorescence, as this time period is insufficient to be transported to late endosomes/lysosomes, based on the DQ-OVA uptake analysis." This only represents an ~ 20% reduction. The major drop in signal is at 40 and 60 min. Coincident with a significant increase in DQ-OVA.

Supplemental figures 6 and 7 seem to be central to the argument. Why are they supplement? Much of the data relies on images of single cells, yet the authors indicate that only ~ 75% of the cells express VE-cadherin. How can we know the cells imaged are the endothelial cells?
Figure 4. – Why was the time course of the mutant-HSA not compared with the WT?

If the traffic is distinct, one would definitely expect to see a difference, with mutant-HSA accumulating in the lysosome and WT being recycled – this seems like a critical experiment.
Figure 7. "From analyzing 36 tubules emerging between 32-40 minutes chase, all were positive for the WT HSA-AF568 signal whereas only 3/36 (8%) were also positive for rHSAH464Q-AF488."
Why not show this data in the figure?

Figure 8 – macropinosome is presumably um not nm.
The numbering text in the first paragraph of the discussion should be updated to remove duplicate number threes.
Please double check the scale bars on S1.C and S1.D – They are both stated to be 50 um.
Figure 1 – white arrows should be described in figure legends for F and G.
Please comment on the lower bands in the mCherry WB in S4A
3B – consider changing labels above figures – this is an interpretation of what you think is happening, not what you did in the experiment (e.g. no HSA added or incubated for X minutes)

First revision

Author response to reviewers' comments

9 June, 2023

Jennifer Lippincott-Schwartz
Editor
Journal Cell Science

Dear Jennifer,

Thank you for your email on 6 March, 2023, regarding the decision on our manuscript (JOCES/2022/260912) entitled "The endosomal system of primary human vascular endothelial cells and albumin-FcRn trafficking". We were pleased that the reviewers were positive with the study and considered the findings "high quality data" and " valuable addition to the field

© 2023. Published by The Company of Biologists under the terms of the Creative Commons Attribution License (https://creativecommons.org/licenses/by/4.0/).

(Reviewer #1) and includes "an important study that contributes to the understanding of serum albumin homeostasis" (Reviewer #2).

We have noted the issues and suggestions raised by the reviewers. In the revision, we have amended the text in response to the concerns of the reviewers and have made minor amendments to four Figures as suggested by the reviewers. Reviewer #2, in particular, was concerned about the balance between the main figures and the supplementary figures. On submission we were limited by the journal policy of 8 main figures and, in addition, we had reached the word limit which impacted on the depth of discussion. We are grateful to the Editor who has agreed to consider exceeding the word and the figure limit to address the concerns of the reviewers.

The alterations in the text of the revised manuscript are highlighted in blue. The responses to the individual comments by the reviewers are as follows:

Reviewer 1:
We thank the reviewer for his/her positive comments stating that "The capacity of BOEC for constitutive macropinocytosis is highly relevant in this context and important to document". The reviewer indicates a few points that should be addressed before publication, as follows:

General comments

1. The data show convincingly and interestingly that FcRn does not transit the classic Rab11 recycling endosomes in BOEC. However, the possible identity of the alternative tubular transport carriers for recycling to the PM (as in Figure 8) is not discussed. How do these structures or the HSA trajectories measured here, compare to the 'fast recycling pathway' from macropinosomes described earlier by these authors and other groups?

The reviewer asks about the identity of the tubular transport carriers for recycling to the PM and comparison with other fast recycling pathways. In the discussion of the original manuscript, we had compared the recycling pathway described in BOECs with our earlier study in primary macrophages, namely the velocities (~ 0.4 µm/s) and morphology (5 – 7 µm long) of FcRn-mCherry and HSA-AF488-positive tubular carriers observed resemble the phenotype of SNX5-positive tubular transport carriers mediating FcRn-dependent recycling of endocytosed HSA in primary BMDMs (Toh et al, J Cell Sci, 2019, 133, jcs235416). Unfortunately, we are unable to localise SNX5 in BOECs as the available antibodies do not cross react with human SNX5. To our knowledge there have been very few other studies examining the fast recycling pathways derived from macropinosomes. In the future it will also be of interest to compare the macropinosome recycling pathway with the SNX Bar- mediated endosomal sorting tubules from early endosomes (Van Weering *et al.*, 2012, EMBO J, 31; 4460-4480). A key feature for comparison will be the precise diameter of the tubules, as assessed by EM, as the diameter of the tubules is defined by the machinery responsible for driving the tubulation. In the revision, we have included this comparison between the recycling tubular carriers of BOECs and primary macrophages and suggestions of future experiments (page 20/21).

2.The internalisation pathway for FcRn is not identified here but conjectured to be (or include) clathrin vesicles. It might be of interest in the future to track FcRn uptake with 10 kDa dextran and to measure intersection with 70 kDa dextran in macropinosomes. This arm of the pathway is less important for the current story than the recycling 'exit' pathway for FcRn and HSA.

Tracking the internalisation pathway of FcRn is a good suggestion by the reviewer for future studies. We do know that the cytoplasmic tail of FcRn has motifs for internalisation by the clathrin pathway (Tesar and Bjorkman, 2010, Curr Opin Struct Biol 20, 226-233). It is also possible FcRn is delivered via the membranes of the macropinosomes and/or from early endosomes. Defining the contributions from these pathways is highly relevant for future studies and will require detailed analyses. One technical challenge related to the suggestion by the reviewer of using fluid phase markers is that 10kDa dextran will be taken up by both pinocytosis and macropincytosis and discrimination between the two endocytic systems could be problematic.

© 2023. Published by The Company of Biologists under the terms of the Creative Commons Attribution License (https://creativecommons.org/licenses/by/4.0/).

Reviewer 1 Comments for the Author:
Minor comments to be addressed.

3.The BOEC cells are presented as a novel feature of the manuscript, and their generation is nicely described in Figure S1 and on page 5. However, it also would be helpful to have more information on the reproducibility of these cell phenotypes. Brief comments about the reproducibility of the cell growth, appearance and endothelial markers in cells derived from different batches of blood/PBMCs , and over different passages, would be helpful.

The reviewer requests additional information on the reproducibility of the primary endothelial cultures. We prepared 4 different batches of BOECs, the first attempt was not successful however the following 3 batches were successful. We used BOEC lines from 2 of these batches, and the cell lines were >94% positive for CD144 at early passages (eg. Passage 5 and 6) and the morphology and growth were similar between the different lines from the two batches. The cell surface levels of CD144 waned gradually with passage number. As an example, one of the lines was 99.7% CD144-positive at P5, 96.7% at P12 and 90.8% at P14. The experiments described in the manuscript used two different cell lines derived from two different donors, and CD144 levels were >90% for all experiments. Additional information has been given in the methods and in the results section to include this information.

4.The text often refers to staining in 'large globular 'or 'globular endosomal structures ' (eg with reference to Figures 6 and 7) in describing the images. It might be less cumbersome and more informative for the reader to simply assign them the labels of macropinosomes or of maturing macropinosomes according to the model in Figure 8. The continuity of the mature macropinosome/early endosome is sufficiently well defined and accepted in the literature.

We appreciate that the reviewer considers that the literature is sufficiently well defined that the we can replace the terms large globular structures' or globular endosomal structures as macropinosomes in relation to the data associated with Figures 6 and 7. In the revision we have now replaced many of the original terms with macropinosomes.

5.The reference cited on pg 11 as Wang et al 2014 does not appear in the list of references.
We thank the reviewer for noting this omission. The Wang et al 2014 reference has now been included in the revision.

6.Label axis on Fig 4D as HSA fluorescence since rest of figure is DQ-OVA
The Y axis of Figure 4D has been re-labelled HSA fluorescence, as requested

7. The discussion should mention or speculate about possible markers for the tubular transport carriers and compare/contrast this with fast recycling pathways in the literature.
In the discussion of the revision, we have expanded the discussion on the possible markers and complexes which may be involved in the formation of the tubular transport carriers based on other fast recycling pathways (page 20/21 of the revision), as follows. *In other cell types, newly formed macropinosomes have also been shown to tubulate extensively, a process dependent on SNX-Bar domain proteins (Kerr et al., 2006) . Additional candidates that may be involved in formation of tubular carriers include SNX17 (McNally et al., 2017; van Kerkhof et al., 2005), SNX27 (Steinberg et al., 2013), Rab4 and the retromer (Cullen and Steinberg,2018) and retriever complexes (McNally et al., 2017).* It would be worthwhile to examine whether any of these components are found on the recycling tubules emerging from macropinosomes in primary endothelial cells.

Reviewer 2:
We thank the reviewer for the positive comments on the importance of the study.

However, in its current form, this study is challenging to read, with many of the key findings relegated to supplemental information. Additionally, the arrangement and display of the data could be substantially improved to focus on the illustrating the key points.

© 2023. Published by The Company of Biologists under the terms of the Creative Commons Attribution License (https://creativecommons.org/licenses/by/4.0/).

The reviewer raises a concern with respect to the allocation of data between the main figures and the supplementary figures. The original manuscript has 8 main figures which is the maximum allowed by *jcs*. In addition, there are also 7 supplementary figures. Reviewer #2 considers some of the supplementary figures should be moved into the main set of figures. The paper covers a number of aspects of cell biology, including stem cell differentiation, characterisation of primary endothelial cells, the pathway of endocytic uptake of albumin and intracellular trafficking of albumin-FcRn. We gave considerable thought as to which figures should be in the main set of figures and which would be supplementary, given the potential broad readership of the paper in endothelial cell biology, macropinocytosis and FcRn ligand recycling. The supplementary figures should be considered a core element of the paper and highly relevant to the story, and not data of lesser value. It was not clear if the reviewer is aware of the figure limitations for *jcs*. Nonetheless we can see the benefit of moving one or two of the supplementary figures into the main set. After consultation with the editor, we were pleased that we are able to exceed the main figure limit and have now moved two of the supplementary figures (Figures S5 and S7), as suggested by the reviewer, into the main set (see points 6 and 12 below). Hopefully this will address the concern raised here by the reviewer.

Reviewer 2 Comments for the Author:
Major concerns:

1.It is difficult to sort out what are bonified macropinosomes versus some other type of early endosomal compartment.
The morphology of the endosomes in figures 1F and 1H appears more consistent with sorting endosomes and docked early endosomes rather than the smooth macropinosomes we are accustomed to seeing in macrophages and cancer cells. Part of this is likely due to fixation and staining strategies. Figure 3D is critical. This would likely be much clearer as a live-cell experiment. In which the dextran and HSA could be visualized early e.g. 5-10 min pulse with dextran + HSA, wash and image for another 10-20 min. Fixation of macropinosome contents is difficult and leads to the punctate morphology on the periphery of the macropinosome. The data in Fig. 3 are consistent with this.

The reviewer asks about the morphology of the macropinosomes/endosomes and makes number of points. The relationship between these points and how it may impact on the interpretation and conclusions of the paper is not clear to us. Our response to these points is as follows:
To clarify, we have defined macropinocytosis by size, the co-localisation with the uptake of 70 kDa dextran, and by the selective inhibition of HSA uptake with amiloride. Hence, the identity of macropinocytosis was well defined, as acknowledged by reviewer #1. Sorting endosomes/recycling endosomes are distinct (and discriminated by Rab11 which is lacking on HSA structures) and finally by the lack of co-localisation with transferrin (Tf).
Macropinosomes will mature and are considered to fuse with (late) endosomes, and this point is discussed further under point 2. However, the precise details of the maturation pathway of macropinosomes does not impact on our conclusions.

Although the reviewer describes the smooth membranes of macropinosomes in macrophages and cancer cells, macropinosomes are actually known to be initially irregular in shape, given they are derived from membrane ruffles and, as membrane is then lost via tubulation, the maturing macropinosomes contracts with a more rigid limiting membrane (J Cell Sci, 2006, 119, 3967-3980). Many publications reveal the irregular shapes of macropinosomes, for example Swanson (J Cell Biol, 1993, 121, 1011-1020), Teasdale (J Cell Sci, 2006, 119, 3967-3980), Stow (Bryant et al 2007, J Cell Sci 120: 1818-1828) and our lab (Toh et al, J Cell Sci, 2019, 133, jcs235416). As the reviewer states, fixation may also impact on their appearance. The reviewer suggests a live cell experiment. Indeed, we have included a live imaging experiment of HSA uptake and lysotracker (Movie 2) following a 15 min pulse. In the revision we have also indicated the live imaging movie earlier in the results section for the reader. Hence, we do not consider additional live imaging is required given the collective data in in the paper.

2.In many cell types like macrophages the 30 min. interval used would be very late - the vast majority of macropinosomes would have already undergone fusion with other early endosomes and had many components trafficked away.

© 2023. Published by The Company of Biologists under the terms of the Creative Commons Attribution License (https://creativecommons.org/licenses/by/4.0/).

The reviewer is correct that in macrophages, the process of macropinosome maturation is fast. However, of particular relevance to our paper, there are differences between cell types and a significant finding in this study is the kinetics of uptake and delivery to lysosomes by primary endothelial cells is slower than macrophages. We have used the 30 min chase period following optimisation experiments as the kinetics of transport are slower in endothelial cells compared with macrophages. This difference in kinetics of maturation is illustrated by the data obtained from the uptake and processing of the self-quenched DQ-OVA molecule in endothelial cells compared with macrophages; whereas our previous study showed that DQ- OVA was degraded within a 10 min of chase period following internalisation by bone marrow-derived macrophages, based on fluorescence at 620 nm (Toh et al 2019, *J Cell Sci* 133, jcs235416), DQ-OVA fluorescence at 620 nm required a 40 min chase period in endothelial cells (Fig. 4A), clearly illustrating major differences in transport kinetics to the late endosomes/lysosomes between the two cell types.

The reviewer suggests the maturation during 30 min may include fusion with early endosomes. Although fusion with endosomes *per se* is considered an integral aspect of macropinsome maturation, it remains unclear whether fusion of maturing macropinosomes occurs selectively with late endosomes, which is the dominant view in the literature (Hewlett et al *J Cell Biol*, 1994, 124, 689-703; Salloum et al 2023, *Biochem J* 480; 335-362) and where degradation would take place, or can occur also with early endosomes as the reviewer is suggesting. An early paper by Colin Watts (*J Cell Biol*, 1994, 124, 689-703) indicated macropinosomes did not fuse with early endosomes in A431 cells, and from my understanding there have been very limited recent studies investigating this possibility in other cell types. Regardless, this is not a question being addressed in this paper and the precise maturation pathway for late endosome/lysosomal delivery does not impact on the conclusions of the paper.

3. The manuscript is unclear as to what compartment FcRn predominantly resides in. It is sometimes called a macropinosome, however, there is no data provided illustrating that a fluid phase marker resides within them. Moreover, they invariably look much larger than the HSA and dextran containing compartment. An experiment clarifying this point would be very helpful. It seems more consistent that FcRn is residing in a particular compartment.

The reviewer queries the location of FcRn in BOECs. Although we have not defined the recycling route of FcRn in BOECs, we have indeed shown that the fluid phase marker, HSA, partially co-localises with exogenously tagged FcRn (**Fig.** 8 in the revision). In addition, we showed that FcRn is largely excluded from Tf-AF488 compartments in the BOECs (**Fig. S5 now Fig 3 in the revision**), which marks predominantly recycling endosomes. Furthermore, FcRn-positive structures, representing endogenous FcRn, co-localized strongly with EEA1 (**Fig. 1G**); there was heterogeneity in the size of FcRn- and EEA1-positive structures, suggesting that FcRn may be located, not only in early macropinosomes, but also in early endosomes. The most relevant finding in our study is the co-location of FcRn and the fluid phase marker HSA from early in the chase periods (now **Fig. 8A** in the revision) rather than the precise trafficking route(s) of FcRn.

4.The tubules appear to be independent of macropinocytosis – as FcRn alone seems to do this constitutively. Does EIPA inhibit tubule formation? Does EIPA disburse FcRn to the cell surface?

The reviewer is correct that the recycling of FcRn via tubules appears to be independent of the albumin ligand, which would be expected. However, we do not agree with the suggestion "tubules appear to be independent of macropinocytosis" and this is not a conclusion which our findings support. An important consideration here is that we are **not** activating macropinocytosis in these primary endothelial cells (BOECs), rather it is a constitutive process under out culture conditions. Thus, with constitutive macropinocytosis and constitutive recycling of FcRn, it would be expected that the tubules are independent of uptake of HSA *per se*. This was also the case for primary macrophages (Toh et al 2019, *J Cell Sci* 133, jcs235416). We have not examined whether EIPA results in a redistribution of FcRn. This is a very good suggestion and would be an excellent experiment for a future study for tracking the endocytosis and itinerary of FcRn (see reviewer #1, point 2)

5.The diagram in Figure 8 needs some careful consideration. From the data provided there is little evidence that FcRn is being brought in on macropinosomes coincident with HSA. Rather it appears that FcRn endosomes interacts increasingly with HSA endosomes at later times 40 min

© 2023. Published by The Company of Biologists under the terms of the Creative Commons Attribution License (https://creativecommons.org/licenses/by/4.0/).

chase (Fig. 6) suggesting it is trafficked there. Figure 7A seems to indicate that only one MP out of many FcRn compartments dots co-localizes with HSA488 early, but this increases over time.

The reviewer queries the Figure model (**Fig. 8**) and the pathway by which FcRn arrives at macropinosome compartment. We did not intend to give the impression that the FcRn transport pathway is defined, and indeed the FcRn pathway was presented in **Fig. 8** of the original manuscript as a dotted line to indicate uncertainly. Fig. 8 of the original manuscript is now **Fig. 10** of the revised manuscript. We have now added question marks along these dotted line pathways to indicate that it is unknown and, also, have indicated in the Figure legend that the pathway of FcRn delivery to macropincosomes is not defined and may also be introduced via delivery from early endosomes or fusion with early endosomes. The data presented in the manuscript indicates that FcRn is located within macropinsomes early in the maturation process, based on the IF image of **Fig. 8A** (5 *min chase, where light green indicates an overlap of red and green).*

6.The description of the results feels like it relies equally on supplemental figures and regular figures to string the story together. This really makes the reader do a lot of work. I would suggest a careful consideration of what data is critical for the story and only relegate truly supplemental information to the supplement. For example, the experiments in Fig. S5 indicating that FcRn is not in the recycling endosome are critical, especially given that the morphology of the compartment does not appear to be that of a typical macropinosome. Yet figure 2 uses an entire page to illustrate the dynamics of a tubule - which are frequently associated with both macropinosomes and sorting endosomes. A little more care in the figure flow would make the manuscript more readable.

The balance of supplementary figures and main figures was discussed above under point 1, reviewer #2. Given the flexibility of the Editor following discussion we have moved the two most essential figures from supplementary into the set of the figures. We are reluctant to re- arrange the existing main figures, as Reviewer #1 considered the article to be very clear; therefore, we wish to maintain the current text and figure structure to ensure the clarity is maintained. Figures S5, which demonstrates that FcRn-mCherry does not reside in the recycling endosomes, and Figure S7 have been moved into the main Figure set in the revision.

7. *Please consider making color overlay using magenta and green to make the work more accessible to color-blind readers. https://.ascb.org/science-news/how-to-make-scientific-figures-accessible-to-readers-with-color-blindness/*
We include single channels for the majority of images in the manuscript, to complement the images showing multiple channels. For red/green channels this provides the ability to assess overlap either from the individual images or the merge, and also maintains the advantage of the green/red signals over magenta.

8. *What about a transwell experiment to show the pumping of HSA from basal to apical surface?*
The transport of HSA by transcytosis was not an objective of this study, rather recycling.

Minor comments:

9. This sentence on page 5 is unclear:
"In this study, we adapted the protocol for the generation of BOECs to meet the requirements for investigating FcRn-albumin cell biology in vascular endothelial cells (Fig. S1B), in particular a protocol which allowed a reliable and fast establishment of endothelial cell lines with high proliferative properties."
Yes, we agree this sentence could be better expressed and have been altered to read as follows:
"…….in contrast we only required smaller cell numbers. Therefore, we adapted the protocol for the rapid establishment of endothelial cell lines to collect relatively small number of cells with high proliferative properties"

10. The MIP representation in Figure 2A doesn't seem to add much and the tubules are difficult to see. I would suggest dropping it.

© 2023. Published by The Company of Biologists under the terms of the Creative Commons Attribution License (https://creativecommons.org/licenses/by/4.0/).

Figure 2A has been altered and only one cell, together with the magnificent is shown. The tubules are clearly indicated by the arrows. We believe, the now reduced set of images in Fig. 2A, is important to show.

11. Figure 4 appears to contradict the interpretation in the results (that recycling is the predominant mode) The DQ-OVA data shows heavy depredation beginning at 40 min. "In particular, the reduction in HSA fluorescence per cell during the first 40 minutes of chase, suggests that recycling may be responsible for the loss of fluorescence, as this time period is insufficient to be transported to late endosomes/lysosomes, based on the DQ-OVA uptake analysis." This only represents an ~ 20% reduction. The major drop in signal is at 40 and 60 min. Coincident with a significant increase in DQ-OVA.

In Fig. 4D (now **Fig. 5D** in the revision) the reduction in the total cell associated HSA-AF488 signal prior to the loss of self-quenched DQ-OVA is indicative of recycling. The data should be taken together with the data shown in Fig. 5, where very little of the HSA fluorescence is found associated with the late endosomes (CD63 positive compartment), a location required for fluorescence of DQ-OVA. The kinetics of recycling will include partitioning of the HSA- FcRn into tubules, fission of tubules and transport of tubular carrier to the cell surface. During these intracellular events the HSA-AF488 will contribute to the total cell fluorescence. Hence the **34.4%** reduction (rather than the 20% reduction stated by the reviewer) in the first 40 min chase (**Fig. 5D**) represents potential HSA already secreted from the cell, and does not take into account the load of HSA segregated into transport carriers which has been diverted from the late/endosome lysosome pathway.

12. Supplemental figures 6 and 7 seem to be central to the argument. Why are they supplement?

See above under point 6. We have moved supplementary Figures S5 and S7 into the main set. However, due to limitations on the number of main figures, supplementary Figure S6 remains as supplementary figure (now supplementary Figure S5 in the revision). This figure, Figure S5, is an extension of Figure 6 in the main figure set. As discussed above, the supplementary figures should be considered a core element of the paper and limitations in number of main figures precludes moving more than 2 figures into the main set.

13. Much of the data relies on images of single cells, yet the authors indicate that only ~ 75% of the cells express VE-cadherin. How can we know the cells imaged are the endothelial cells?

We used BOEC populations that expressed >90% CD144 and often >95% expression. Figure 1 is a FACS plot which clearly shows >90% of BOECs express CD144 and also >95% vWF. In the original manuscript we stated that cultures were discarded once CD144 dropped below 75%. However, we only used BOECs in experiments which expressed CD144 >90%. We acknowledge this information in the original manuscript was incomplete and thank the reviewer for highlighting the issue. We have expanded the information on the CD144 level of expression with cell passages and on the reproducibility of BOEC generation, on page 6 of the revision and on page 25 in the methods. See further details also under point 3 of reviewer #1.

14. Figure 4. - Why was the time course of the mutant-HSA not compared with the WT? If the traffic is distinct, one would definitely expect to see a difference, with mutant-HSA accumulating in the lysosome and WT being recycled - this seems like a critical experiment.

Figure 4 was focused on the relationship between the reduction of the WT HSA and delivery to degradative compartments. The subsequent experiments then went on to compare the location of WT and mutant HSA at the 40 min and 60 min chase periods by IF (Fig. 6) and live imaging of the mutant HSA with FcRn-mCherry (Fig. 8) and live imaging with both mutant and WT HSA together (Fig. 9). We considered that the comparison of the location of the wild-type and mutant HSA during the time course to be a critical experiment to assess the level of mutant HSA accumulating in the lysosome and the selective recruitment of WT but not mutant HSA in tubular structures. These experiments did indeed show that the mutant HSA accumulated in the lysosome.

15. Figure 7. "From analyzing 36 tubules emerging between 32-40 minutes chase, all were positive for the WT HSA-AF568 signal whereas only 3/36 (8%) were also positive for rHSAH464Q-AF488." Why not show this data in the figure?

© 2023. Published by The Company of Biologists under the terms of the Creative Commons Attribution License (https://creativecommons.org/licenses/by/4.0/).

We do not think it necessary to have a figure dedicated to this data. Providing the data in the text should suffice and will save figure space.

16. Figure 8 - macropinosome is presumably um not nm.
Thank you for pointing out this error. Yes, the diameter should be μm.

17. The numbering text in the first paragraph of the discussion should be updated to remove duplicate number threes.
Thank you for pointing out this duplication, which has been corrected in the revision.

18. Please double check the scale bars on S1.C and S1.D - They are both stated to be 50 μm.
Thank you for pointing this out. The scale for S1.D represents 25 microns, rather than 50 microns. This has been corrected in the revision.

19. Figure 1 - white arrows should be described in figure legends for F and G.
Thank you for pointing out this omission. The white arrows have now been described in the legends of Fig. 1F and G.

20. Please comment on the lower bands in the mCherry WB in S4A
The lower band of ~30 kDa in the mCherry WB in S4A is the size of free mCherry and is likely due to the cleavage of mCherry from the fusion protein either within the cell or upon cell extraction. These mCherry fragments will be soluble and would be diffusely distributed within the cytosol and would represent minor fluorescent signals in the IF images. A comment has been included in the text of the revision on page 9.

21. 3B - consider changing labels above figures - this is an interpretation of what you think is happening, not what you did in the experiment (e.g. no HSA added or incubated for X minutes)
Good point. Yes, the labels above the figures have been changed to indicate the treatment. (now **Fig. 4B** in the revision).

We thank the reviewers for their suggestions on our manuscript and look forward to a decision on our revised manuscript.

Yours sincerely,

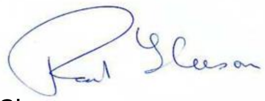

Paul Gleeson

Second decision letter

MS ID#: JOCES/2022/260912

MS TITLE: The endosomal system of primary human vascular endothelial cells and albumin-FcRn trafficking

AUTHORS: Andreas Pannek, Janine Becker-Gotot, Steven K Dower, Anne M Verhagen, and Paul A. Gleeson

ARTICLE TYPE: Research Article

I am happy to tell you that your manuscript has been accepted for publication in Journal of Cell Science, pending standard ethics checks.

© 2023. Published by The Company of Biologists under the terms of the Creative Commons Attribution License (https://creativecommons.org/licenses/by/4.0/).
